# Supplementary material for: Tisp40 prevents cardiac ischemia/reperfusion injury through the hexosamine biosynthetic pathway in male mice
Source: Nat Commun. 2023 Jun 8;14:3383. doi: 10.1038/s41467-023-39159-0 (PMC10250363; doi:10.1038/s41467-023-39159-0)

Supplemental Information

Supplementary Table 1. List of the primary antibodies used for western blot (WB), immunofluorescence (IF) staining, immunohistochemical (IHC) staining and ChIP assay in this study.

Antibody	Source	Catalog No.	Application (dilution)
α-actinin	Abcam	ab68167	IF (1:100)
α-SMA	Abcam	ab5694	IHC (1:100)
BAX	CST	2772	WB (1:1000)
BCL-2	Abcam	ab196495	WB (1:1000)
GAPDH	CST	2118	WB (1:1000)
GFPT1	Proteintech	14132-1-AP	WB (1:500), IF (1:100)
HA-Tag	CST	3724	WB (1:2000), IF (1:200), ChIP (1:50)
KDEL	Abcam	ab176333	IF (1:100)
Lamin B1	Abcam	ab16048	WB (1:1000)
O-GlcNAc (CTD110.6)	CST	9875	WB (1:1000), IF (1:100)
Tisp40	Santa Cruz	sc-390842	WB (1:500), IF (1:100)
p-p65	CST	3033	WB (1:1000)
t-p65	CST	8242	WB (1:1000)

Supplementary Table 2. Primer sets used for quantitative real-time polymerase chain reaction (qPCR), promoter cloning and ChIP assay in this study.

Gene	Species	Primer sequence (5′→3′)		Application
α-Mhc	Mouse	Forward	GTCCAAGTTCCGCAAGGT	qPCR
		Reverse	AGGGTCTGCTGGAGAGGTTA	
β-Mhc	Mouse	Forward	CCGAGTCCCAGGTCAACAA	qPCR
		Reverse	CTTCACGGGCACCCTTGGA	
Anp	Mouse	Forward	ACCTGCTAGACCACCTGGAG	qPCR
		Reverse	CCTTGGCTGTTATCTTCGGTACCGG	
Col1α1	Mouse	Forward	AGGCTTCAGTGGTTTGGATG	qPCR
		Reverse	CACCAACAGCACCATCGTTA	
Col3α1	Mouse	Forward	CCCAACCCAGAGATCCCATT	qPCR
		Reverse	GAAGCACAGGAGCAGGTGTAGA	
Creb3l1	Mouse	Forward	CCTTGTGCCTGTCAAGATGGAG	qPCR
		Reverse	GCAGCAGCCATGGCAGAGGAG	
Creb3l2	Mouse	Forward	GGGCTGGAGTTGGTCATTTTT	qPCR
		Reverse	TGCAAAGTATCCAAACCTGACTGT	
Creb3l3	Mouse	Forward	CAGCTCAAGAAAGCAGGAAG	qPCR
		Reverse	AGCTGCTCCAGAAGAGACAA	
Creb3l4	Mouse	Forward	GAGCTGGGATTCAACGGTCC	qPCR
		Reverse	CATAGACAACCTCATAGAGGGCA	
Ctgf	Mouse	Forward	TGACCCCTGCGACCCACA	qPCR
		Reverse	TACACCGACCCACCGAAGACACAG	
Gapdh	Mouse	Forward	ACTCCACTCACGGCAAATTC	qPCR
		Reverse	TCTCCATGGTGGTGAAGACA	
Gapdh	Rat	Forward	GACATGCCGCCTGGAGAAAC	qPCR
		Reverse	AGCCCAGGATGCCCTTTAGT	
Gfpt1	Mouse	Forward	TAAGGAGATCCAGCGGTGTC	qPCR
		Reverse	CAGCTGTCTCGCCTGATTGA	

Gfpt1	Mouse	Forward	GCTAGCGTGGCAAGGATGTGATGTGG	Promoter cloning
		Reverse	CTCGAGTGGCAATGAGAGGACTCTGG	
Gfpt1	Mouse	Forward	CTCTGCACACTGACACGTCTG	ChIP
		Reverse	GTGGCAATGAGAGGACTCTGG	
Gfpt1	Rat	Forward	AGTTGGCACAAGGCGAGGTA	qPCR
		Reverse	ACGGCACTTGCATCAGAAGC	
Gfpt2	Mouse	Forward	AGCCATCCAGACCTTGCAGA	qPCR
		Reverse	CACAATGAGCCTTCGGCATC	
Gfpt2	Rat	Forward	CCACAGCGTTCAGACAAAGA	qPCR
		Reverse	CTCAAACTCGTAGCCCTTGC	
Gnpnat1	Mouse	Forward	AGAAGTGGACTGGAGTCAGA	qPCR
		Reverse	GGTCACATCTTCCACAACTG	
Lox	Mouse	Forward	CAGAGGAGAGTGGCTGAAGG	qPCR
		Reverse	CCAGGACTCAATCCCTGTGT	
Oga	Mouse	Forward	AGCGAAGATGGCAGAGGAGT	qPCR
		Reverse	CCGTGCTCGTAAGGAAGGTA	
Oga	Rat	Forward	CCACACCCTTGCCACTT	qPCR
		Reverse	CACCACGTCCTTCCTCAC	
Ogt	Mouse	Forward	GAGTGAAGGTGATGGCGGAA	qPCR
		Reverse	ATGGCCTGAATAGGAGCTGG	
Ogt	Rat	Forward	CCTTCCCCAGAACCGTAT	qPCR
		Reverse	CCAGAGAACATCCATCCCT	
Pgm3	Mouse	Forward	TGAGAGATGCTGCTCCTTCG	qPCR
		Reverse	CTTCTTCAAGGTCCCGCGTG	
Tgf-β1	Mouse	Forward	TGCGCTTGCAGAGATTAAAA	qPCR
		Reverse	CGTCAAAAGACAGCCACTCA	
Uap1	Mouse	Forward	ACCTGCTGCAGTTCTGGAATG	qPCR
		Reverse	CAGCTGCTCTTGATCTCTGGT	

Supplementary Table 3. Serum levels of glycometabolic parameters in Tisp40 WT, KO, NTG and cTG mice.

Genotype	WT (n=6)	KO (n=6)	NTG (n=6)	cTG (n=6)
Fasting blood glucose (mmol/L)	5.22 ±0.73	5.02 ±0.85	5.45 ±0.78	5.47 ±0.56
Insulin (mIU/L)	8.40 ±0.80	8.17 ±0.68	8.82 ±1.16	8.38 ±0.88
HOMA-IR (mmol/L × mIU/L)	1.93 ±0.19	1.82 ±0.32	2.12 ±0.34	2.04 ±0.36
Triglyceride (mmol/L)	0.33 ±0.07	0.34 ±0.08	0.32 ±0.06	0.35 ±0.11
Total cholesterol (mmol/L)	2.07 ±0.35	2.10 ±0.46	2.27 ±0.41	2.45 ±0.55

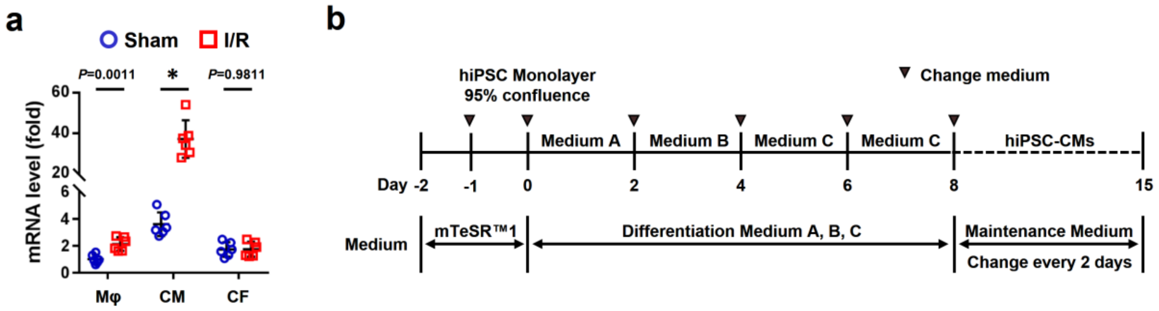

Supplementary Figure 1. Tisp40 expression and nuclear translocation are induced by cardiac I/R injury. **a**, Mice received sham or cardiac ischemia/reperfusion (I/R) surgery (ischemia for 45 min and reperfusion for 24 h) with cardiac macrophages (Mφ), cardiomyocytes (CM) and cardiac fibroblasts (CF) isolated, and then quantitative real-time PCR was performed for the analysis of *Creb3l4* mRNA level in these cells (n=6). **b**, Protocol diagram for the induction and culture of human-induced pluripotent stem cell-derived cardiomyocytes (hiPSC-CMs). All data are expressed as the mean ± standard deviation (S.D.), and analyzed using an unpaired two-tailed Student′s *t*-test. *$P < 0.0001$. Source data are provided as a Source Data file.

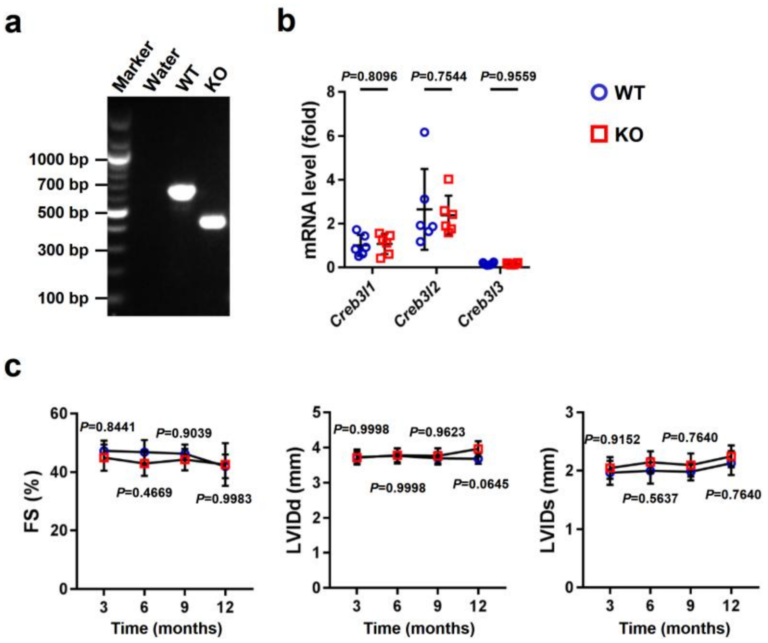

Supplementary Figure 2. Tisp40 deficiency does not affect cardiac function at baseline. a, Genotyping results of Tisp40 knockout (KO) mice and the wild type (WT) littermates (n=6). **b**, Heart samples of Tisp40 KO mice or WT littermates were exposed to quantitative real-time PCR for the analysis of *Creb3l1*, *Creb3l2* and *Creb3l3* mRNA levels (n=6). **c**, Cardiac function of Tisp40 KO mice or WT littermates was analyzed by transthoracic echocardiography at the indicated time points, and presented as fractional shortening (FS), left ventricle internal diameters at diastole (LVIDd) or systole (LVIDs) (n=6). All data are expressed as the mean ±S.D., and analyzed using an unpaired two-tailed Student′s *t*-test (figure S2b) or repeated measures analysis of variance (ANOVA) followed by Sidak post hoc test (figure S2c). Source data are provided as a Source Data file.

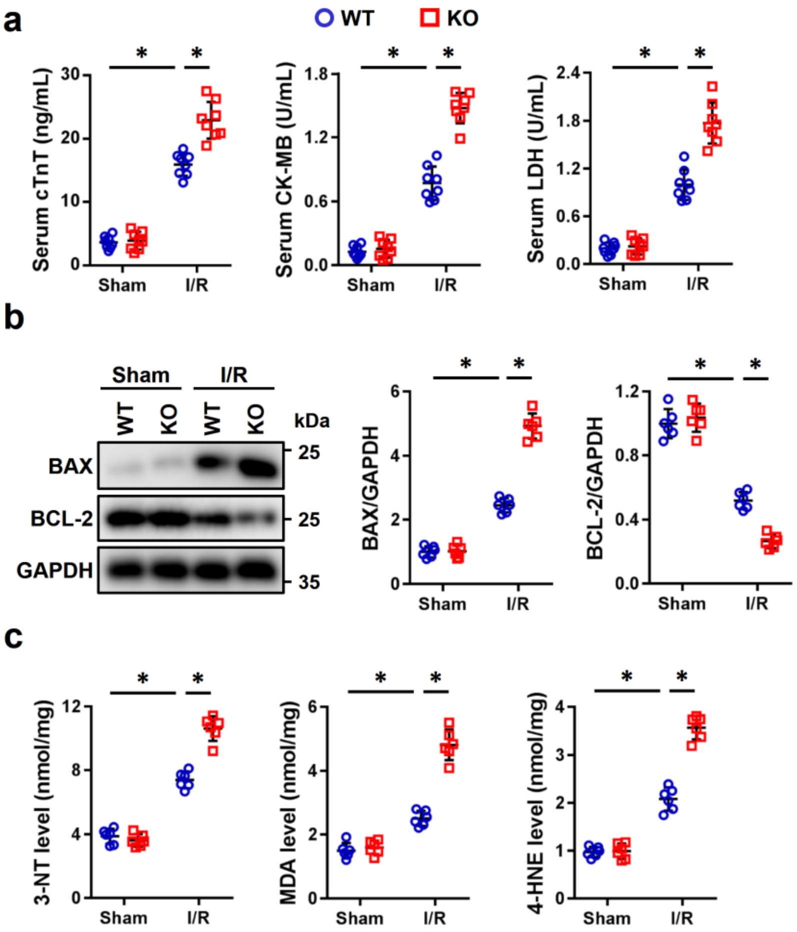

Supplementary Figure 3. Tisp40 deficiency exacerbates oxidative stress, apoptosis and cardiac I/R injury in vivo. a, Circulating levels of cardiac isoform of tropnin T (cTnT), creatine kinase isoenzymes (CK-MB) and lactate dehydrogenase (LDH) in Tisp40 KO mice and WT littermates 4 h after I/R surgery (n=6). **b**, Heart samples were collected for western blot 24 h after I/R surgery (n=6). **c**, Levels of 3-nitrotyrosine (3-NT), malondialdehyde (MDA) and 4-hydroxynonenal (4-HNE) in the heart (n=6). All data are expressed as the mean ± S.D., and analyzed using one-way ANOVA followed by Tukey post hoc test. **P* < 0.0001. Source data are provided as a Source Data file.

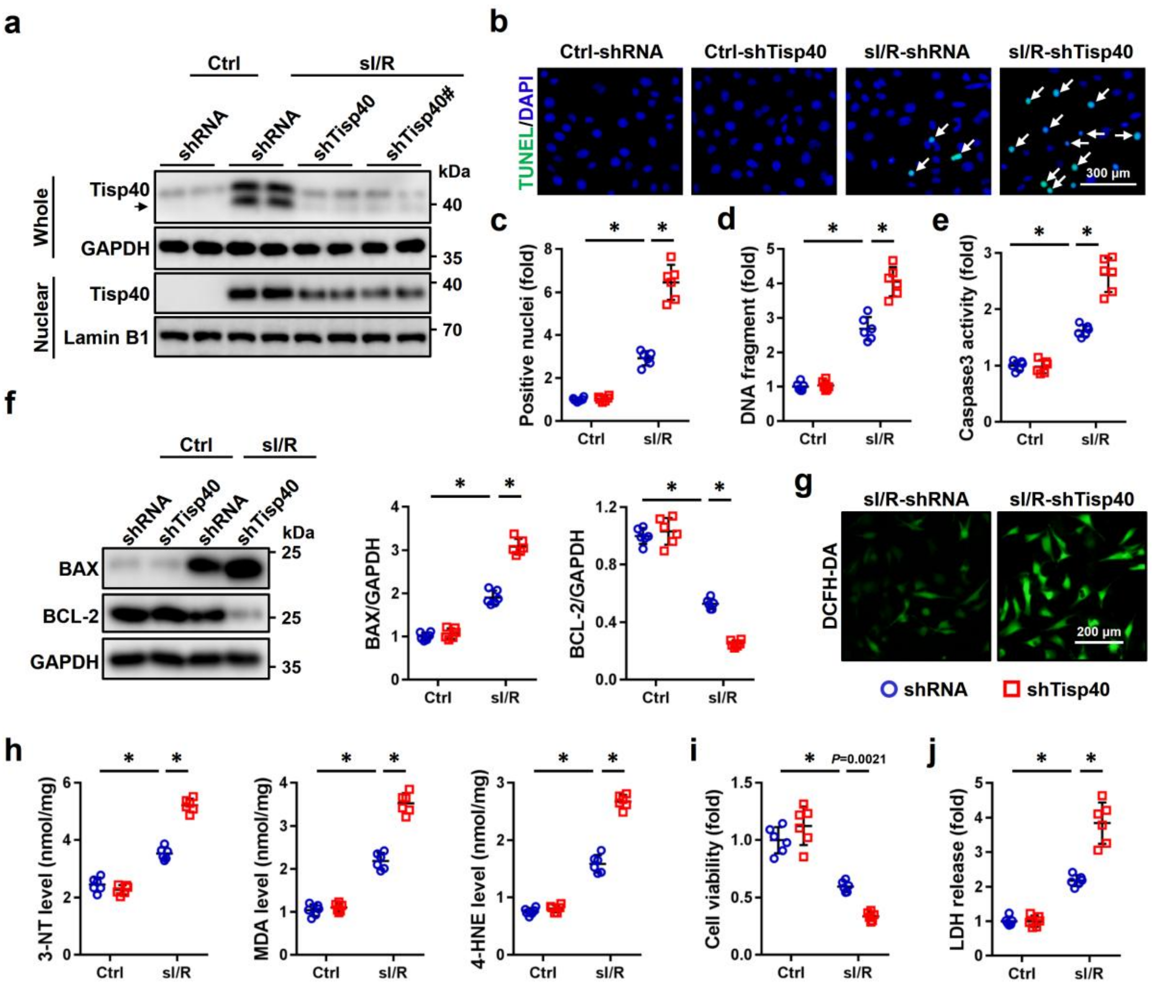

Supplementary Figure 4. Tisp40 knockdown by shTisp40 exacerbates sI/R-induced oxidative stress and cardiomyocyte apoptosis in vitro. a, Neonatal rat cardiomyocytes (NRCMs) were pre-infected with shTisp40 or shTisp40# for 4 h, incubated for an additional 48 h, and then exposed to ischemia for 4 h followed by overnight reperfusion. Next, whole cell lysates and nuclear lysates were prepared for western blot (n=6). **b-c**, Representative TdT-mediated dUTP nick end-labeling (TUNEL) staining images of cell coverslips and quantitative results (n=6). **d**, DNA fragments in NRCMs (n=6). **e**, Caspase3 activity in NRCMs (n=6). **f**, NRCMs were collected for western blot (n=6). **g**, Representative 2′,7′-dichlorodihydrofluorescein diacetate (DCFH-DA) staining images of cell coverslips (n=6). **h**, Levels of 3-NT, MDA and 4-HNE in NRCMs (n=6). **i**, Relative cell viability (n=6). **j**, LDH releases were calculated as (LDH level in ischemia medium + LDH in reperfusion medium)/(LDH in ischemia medium + LDH in reperfusion medium + LDH in cell lysate) (n=6). All data are expressed as the mean ± S.D., and analyzed using one-way ANOVA followed by Tukey post hoc test. *$P < 0.0001$. Source data are provided as a Source Data file.

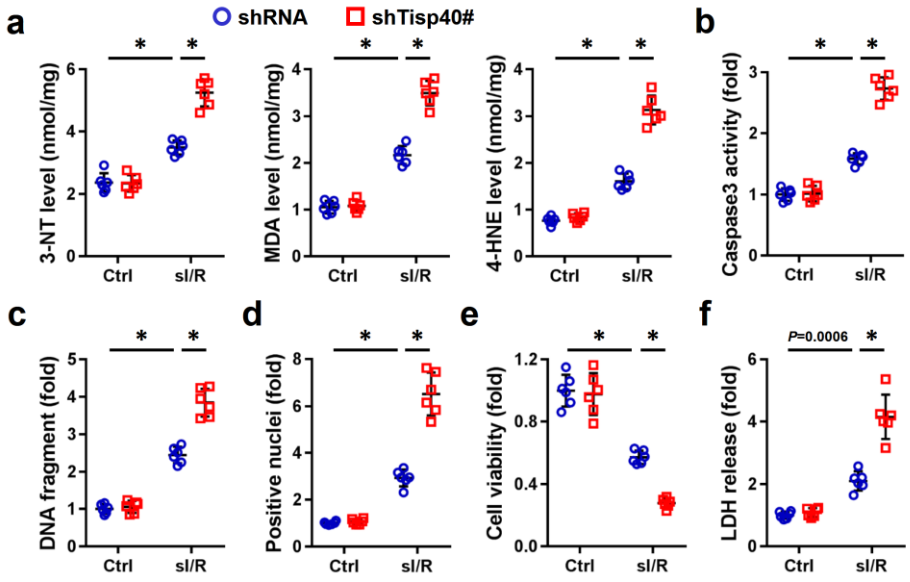

Supplementary Figure 5. Tisp40 knockdown by shTisp40# exacerbates sI/R-induced oxidative stress and cardiomyocyte apoptosis in vitro. a, Levels of 3-NT, MDA and 4-HNE in NRCMs (n=6). **b**, Caspase3 activity in NRCMs (n=6). **c**, DNA fragments in NRCMs (n=6). **d**, Quantitative results of TUNEL staining (n=6). **e**, Relative cell viability (n=6). **f**, LDH releases were calculated as (LDH level in ischemia medium + LDH in reperfusion medium)/(LDH in ischemia medium + LDH in reperfusion medium + LDH in cell lysate) (n=6). All data are expressed as the mean ± S.D., and analyzed using one-way ANOVA followed by Tukey post hoc test. *$P < 0.0001$. Source data are provided as a Source Data file.

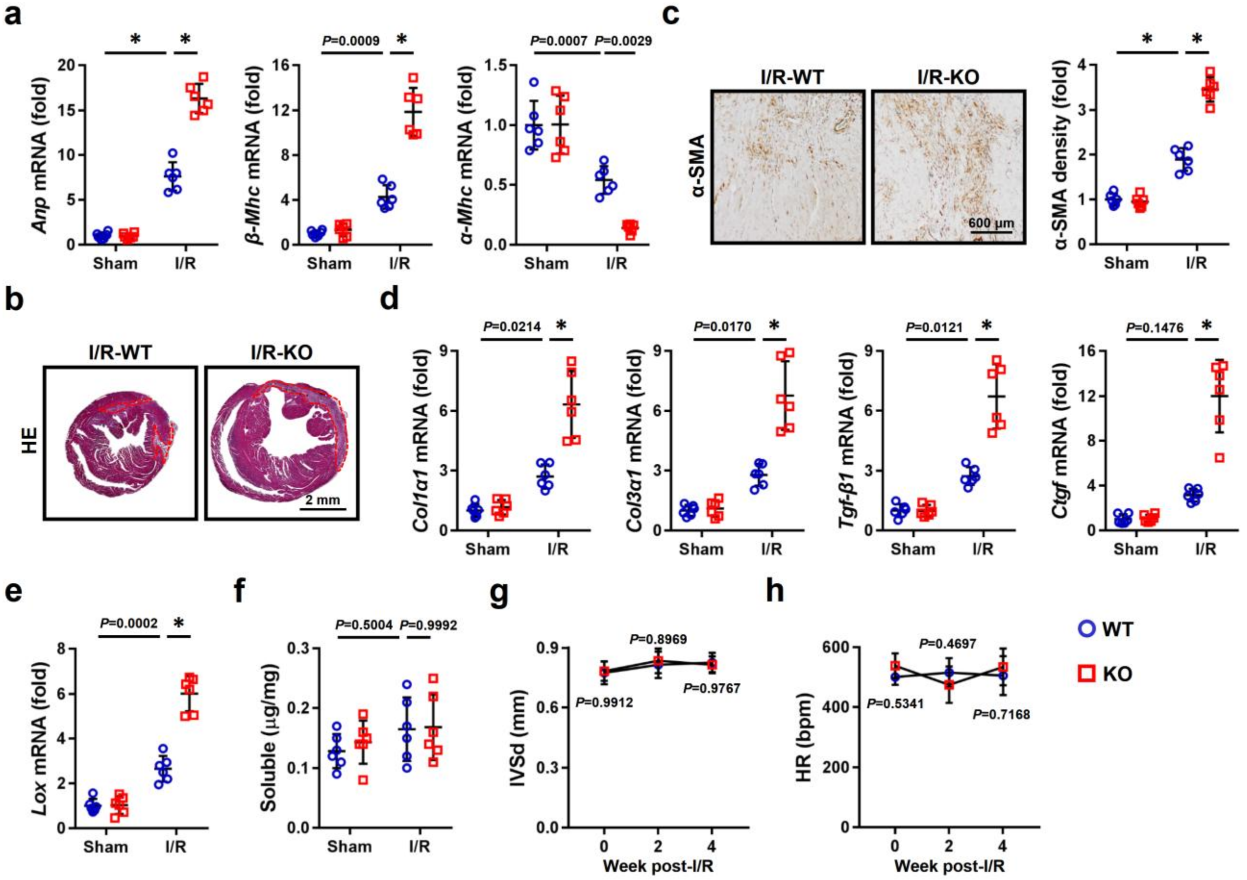

Supplementary Figure 6. Tisp40 deficiency aggravates cardiac remodeling and dysfunction following I/R injury. a, Levels of atrial natriuretic peptide (*Anp*), β-myosin heavy chain (*β-Mhc*) and *α-Mhc* mRNA in the heart 4 weeks post-I/R surgery (n=6). **b**, Heart samples were collected for hematoxylin-eosin (HE) staining to quantify the scar size 4 weeks post-I/R surgery (n=6). **c**, Heart samples were collected for immunohistochemical staining to analyze α-smooth muscle actin (α-SMA) expression 4 weeks post-I/R surgery to evaluate myofibroblast activation (n=6). **d-e**, Levels of collagen 1α1 (*Col1α1*), *Col3α1*, transforming growth factor-β1 (*Tgf-β1*), connective tissue growth factor (*Ctgf*) and lysyl oxidase (*Lox*) mRNA in the heart 4 weeks post-I/R surgery (n=6). **f**, Soluble collagen content in the heart 4 weeks post-I/R surgery (n=6). **g-h**, Cardiac function of Tisp40 KO mice or WT littermates was analyzed by transthoracic echocardiography at the indicated time points, and presented as interventricular septal thickness at diastole (IVSs) and heart rate (HR) (n=6). All data are expressed as the mean ± S.D., and analyzed using one-way ANOVA followed by Tukey post hoc test. For the analysis in figure S6g and h, repeated measures ANOVA followed by Sidak post hoc test was conducted. **P* < 0.0001. Source data are provided as a Source Data file.

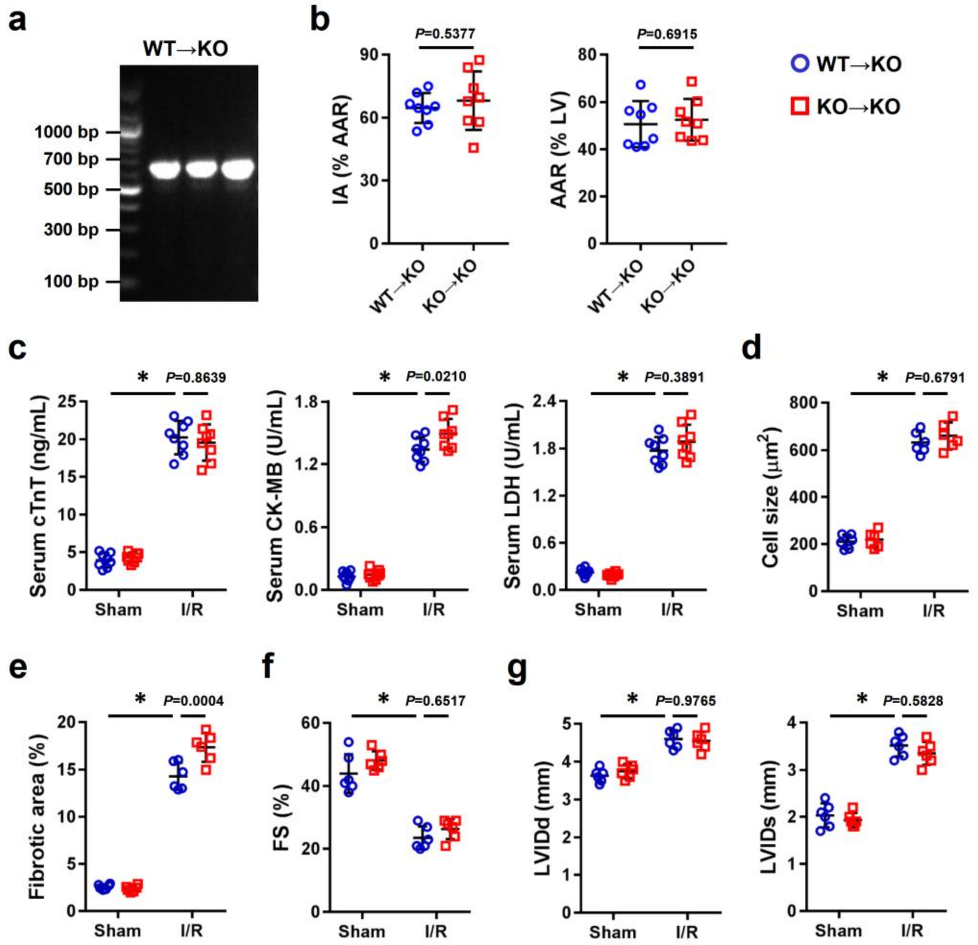

Supplementary Figure 7. Macrophage Tisp40 does not affect cardiac I/R injury. a, Irradiated Tisp40 KO mice were reconstituted with either WT or KO bone marrows, kept for 4 weeks and then peripheral blood was collected for the PCR analysis of hematologic chimerism (n=6). **b**, The relative ratios of infarct area (IA, pale) to the area at risk (AAR, not blue) and AAR to left ventricles (LV) in Tisp40 KO mice reconstituted with either WT or KO bone marrows 24 h after I/R surgery (n=8). **c**, Circulating levels of cardiac isoform of tropnin T (cTnT), creatine kinase isoenzymes (CK-MB) and lactate dehydrogenase (LDH) in Tisp40 KO mice reconstituted with either WT or KO bone marrows 4 h after I/R surgery (n=6). **d-e**, Quantitative results of the cross-sectional area of cardiomyocyte and collagen deposition 4 weeks post-I/R surgery (n=6). **f-g**, Cardiac function of Tisp40 KO mice reconstituted with either WT or KO bone marrows 4 weeks post-I/R surgery (n=6). All data are expressed as the mean ± S.D., and analyzed using one-way ANOVA followed by Tukey post hoc test. For the analysis in figure S7b, an unpaired two-tailed Student′s *t*-test was conducted. **P* < 0.0001. Source data are provided as a Source Data file.

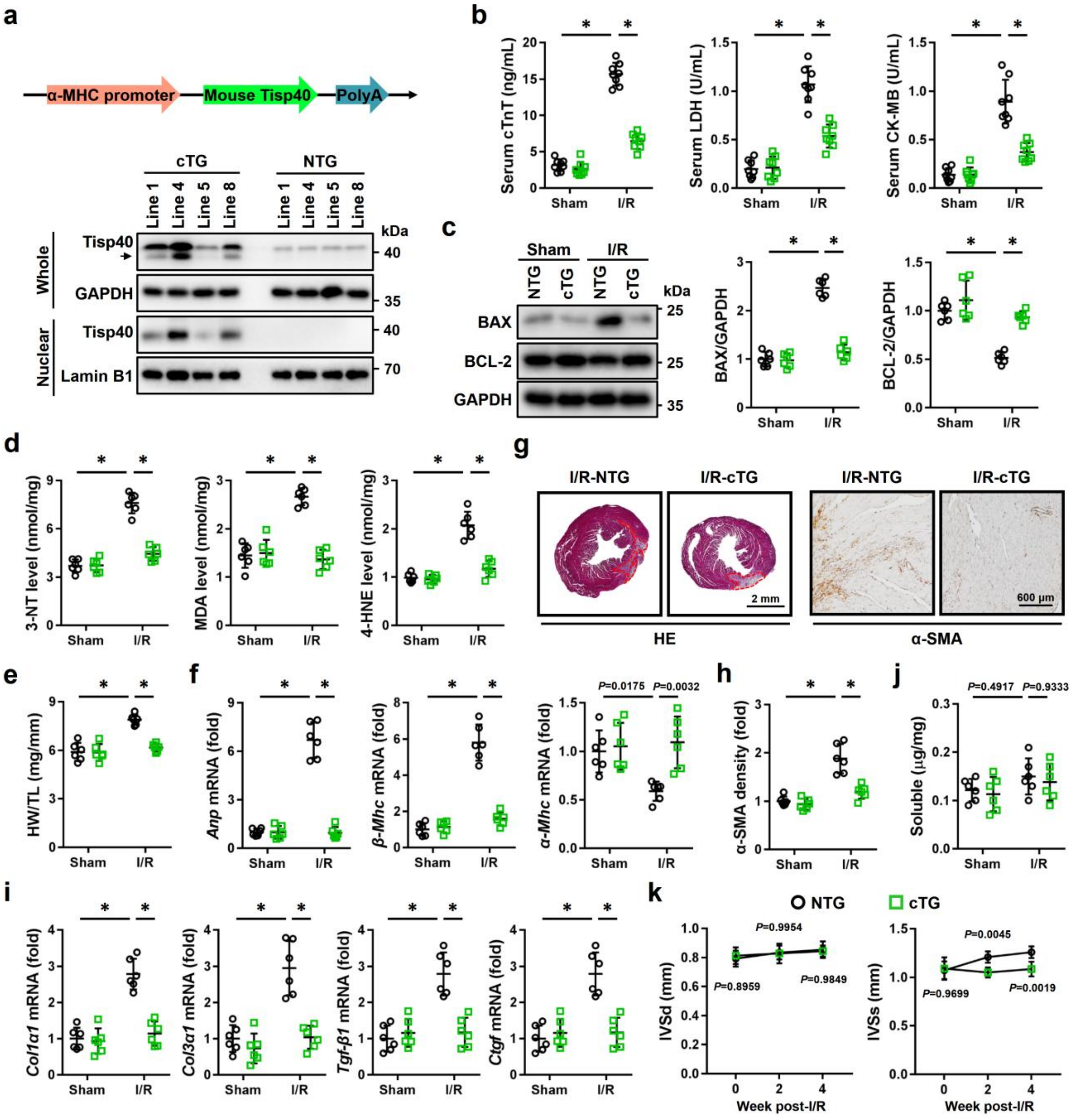

Supplementary Figure 8. Cardiomyocyte-specific overexpression of full-length Tisp40 prevents I/R-induced acute cardiac injury, remodeling and dysfunction in mice. a, Schematic diagram illustrating the construct used to generate cardiomyocyte-restricted Tisp40 transgenic (cTG) mice, and the efficiency was confirmed by western blot in Tisp40 cTG mice compared with the matched non-transgenic (NTG) littermates (n=6). **b**, Circulating levels of cTnT, CK-MB and LDH in Tisp40 cTG mice and NTG littermates 4 h after I/R surgery (n=6). **c**, Heart samples were collected for western blot 24 h after I/R surgery (n=6). **d**, Levels of 3-NT, MDA and 4-HNE in the heart (n=6). **e**, Quantitative results of heart weight/tibial length (HW/TL) 4 weeks post-I/R surgery (n=6). **f**, Levels of *Anp*, *β-Mhc* and *α-Mhc* mRNA in the heart 4 weeks post-I/R surgery (n=6). **g**,

Heart samples were collected for HE staining to quantify the scar size, and for immunohistochemical staining to analyze α-SMA expression 4 weeks post-I/R surgery (n=6). **h**, Quantitative results of α-SMA expression to evaluate myofibroblast activation (n=6). **i**, Levels of *Col1α1*, *Col3α1*, *Tgf-β1* and *Ctgf* mRNA in the heart 4 weeks post-I/R surgery (n=6). **j**, Soluble collagen content in the heart 4 weeks post-I/R surgery (n=6). **k**, Cardiac function of Tisp40 cTG mice or NTG littermates was analyzed by transthoracic echocardiography at the indicated time points, and presented as interventricular septal thickness at diastole (IVSd) or systole (IVSs) (n=6). All data are expressed as the mean ± S.D., and analyzed using one-way ANOVA followed by Tukey post hoc test. For the analysis in figure S8k, repeated measures ANOVA followed by Sidak post hoc test was conducted. *$P < 0.0001$. Source data are provided as a Source Data file.

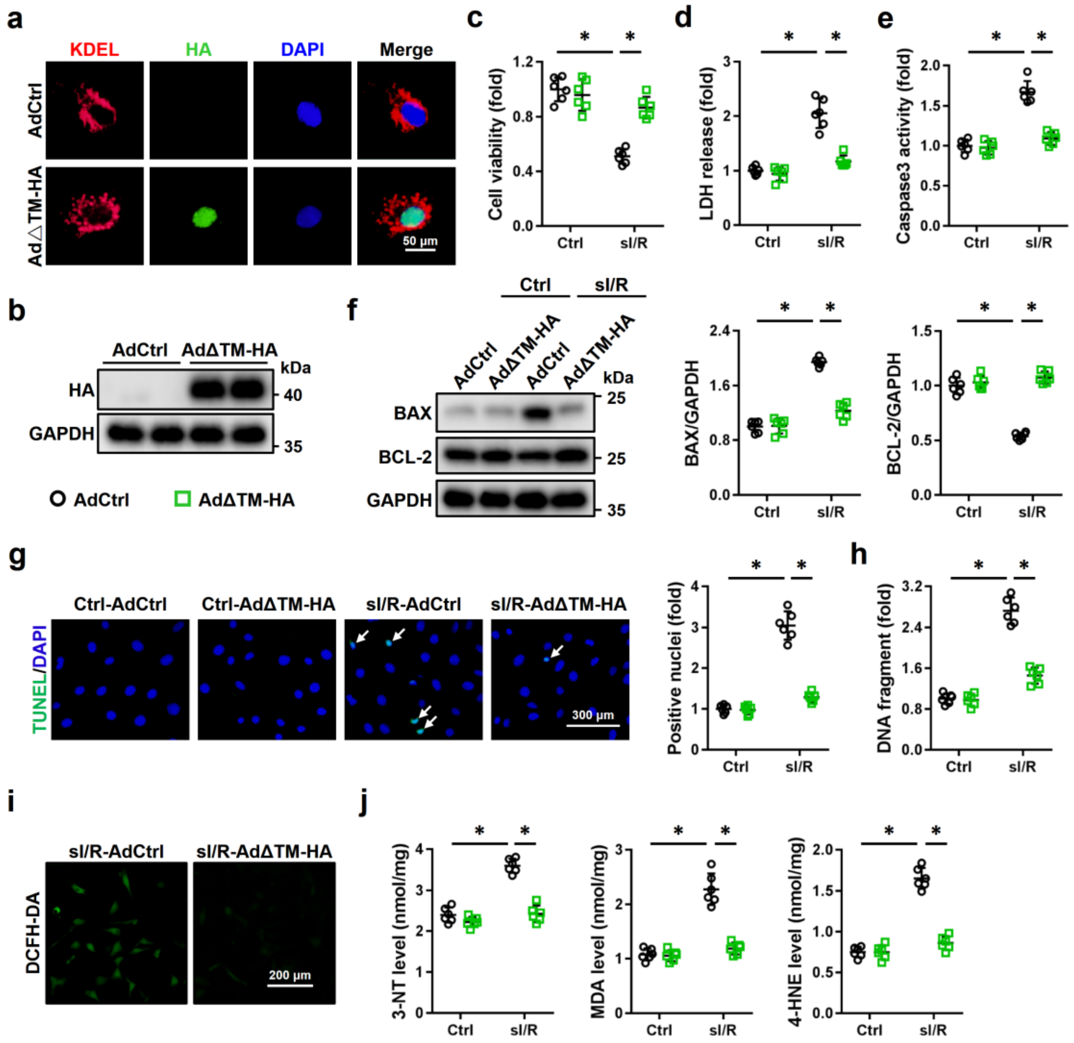

Supplementary Figure 9. Overexpression of nuclear Tisp40 is sufficient to attenuate sI/R-induced oxidative stress and cardiomyocyte apoptosis in vitro. a, NRCMs were infected with AdΔTM-HA or AdCtrl for 4 h, incubated for an additional 48 h, and then were stained with Lys-Asp-Glu-Leu (KDEL, an ER marker, red) and HA (green) (n=6). **b**, NRCMs with AdΔTM-HA or AdCtrl infection were maintained in fresh medium for 48 h, and then were exposed to ischemia for 4 h followed by overnight reperfusion. Next, whole cell lysates were prepared for western blot (n=6). **c**, Relative cell viability (n=6). **d**, LDH releases were calculated as (LDH level in ischemia medium + LDH in reperfusion medium)/(LDH in ischemia medium + LDH in reperfusion medium + LDH in cell lysate) (n=6). **e**, Caspase3 activity in NRCMs (n=6). **f**, NRCMs were collected for western blot (n=6). **g**, Representative TUNEL staining images of cell coverslips and quantitative results (n=6). **h**, DNA fragments in NRCMs (n=6). **i**, Representative DCFH-DA staining images of cell coverslips (n=6). **j**, Levels of 3-NT, MDA and 4-HNE in NRCMs (n=6). All data are expressed as the mean ± S.D., and analyzed using one-way ANOVA followed by Tukey post hoc test. *P < 0.0001. Source data are provided as a Source Data file.

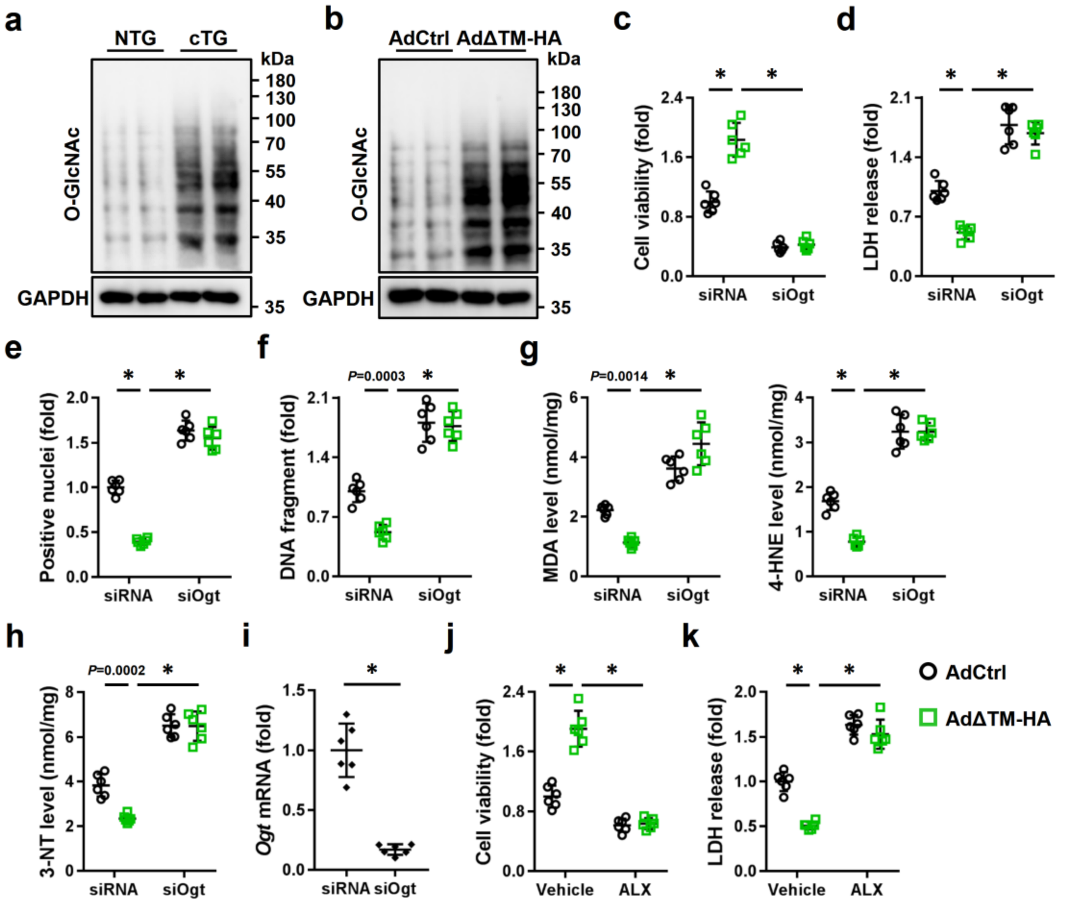

Supplementary Figure 10. OGT inhibition blocks Tisp40 overexpression-mediated cellular protection against sI/R insult in vitro. a, Protein O-GlcNAc levels in Tisp40 cTG or NTG hearts at baseline were evaluated by western blot (n=6). **b**, Protein O-GlcNAc levels in NRCMs with AdΔTM-HA or AdCtrl infection at baseline were evaluated by western blot (n=6). **c**, To knock down endogenous OGT, NRCMs were pre-transfected with siOgt or siRNA for 4 h, followed by the incubation in fresh medium for an additional 24 h before Tisp40 manipulation. Next, cell viability was evaluated (n=6). **d**, LDH releases were calculated as (LDH level in ischemia medium + LDH in reperfusion medium)/(LDH in ischemia medium + LDH in reperfusion medium + LDH in cell lysate) (n=6). **e**, Quantitative results of TUNEL staining in NRCMs (n=6). **f**, DNA fragments in NRCMs (n=6). **g-h**, Levels of 3-NT, MDA and 4-HNE in NRCMs (n=6). **i**, NRCMs transfected with siOgt or siRNA were collected for the analysis of *Ogt* mRNA level (n=6). **j**, To inhibit OGT activity, ALX was added to the medium during reperfusion, and then cell viability was evaluated (n=6). **k**, LDH releases were calculated as (LDH level in ischemia medium + LDH in reperfusion medium)/(LDH in ischemia medium + LDH in reperfusion medium + LDH in cell lysate) (n=6). All data are expressed as the mean ± S.D., and analyzed using one-way ANOVA followed by Tukey post hoc test. For the analysis in figure S10i, an unpaired two-tailed Student′s *t*-test was conducted. *$P < 0.0001$. Source data are provided as a Source Data file.

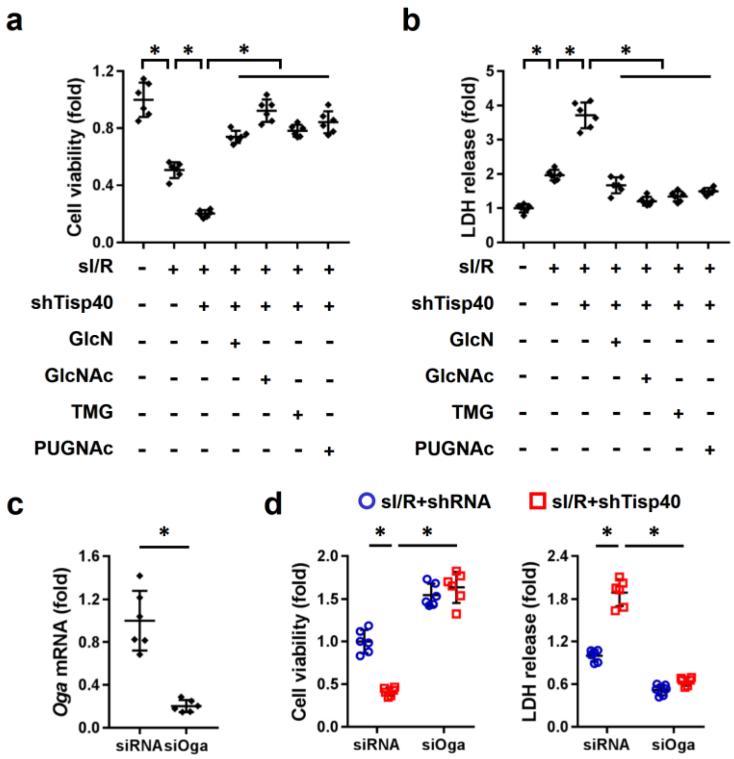

Supplementary Figure 11. OGA inhibition attenuates sI/R-induced cellular damage in Tisp40-silenced NRCMs in vitro. a, Tisp40-silenced NRCMs were treated with TMG or PUGNAc during reperfusion to inhibit OGA, whereas either GlcN or GlcNAc was added to activate hexosamine biosynthesis during reperfusion. Next, cell viability was evaluated (n=6). **b**, LDH releases were calculated as (LDH level in ischemia medium + LDH in reperfusion medium)/(LDH in ischemia medium + LDH in reperfusion medium + LDH in cell lysate) (n=6). **c**, NRCMs transfected with siOga or siRNA were collected for the analysis of *Oga* mRNA level (n=6). **d**, To knock down endogenous OGA, NRCMs were pre-transfected with siOga or siRNA for 4 h, followed by the incubation in fresh medium for an additional 24 h before Tisp40 manipulation. Next, cell viability and LDH releases were evaluated (n=6). All data are expressed as the mean ± S.D., and analyzed using one-way ANOVA followed by Tukey post hoc test. For the analysis in figure S11c, an unpaired two-tailed Student′s *t*-test was conducted. *P < 0.0001. Source data are provided as a Source Data file.

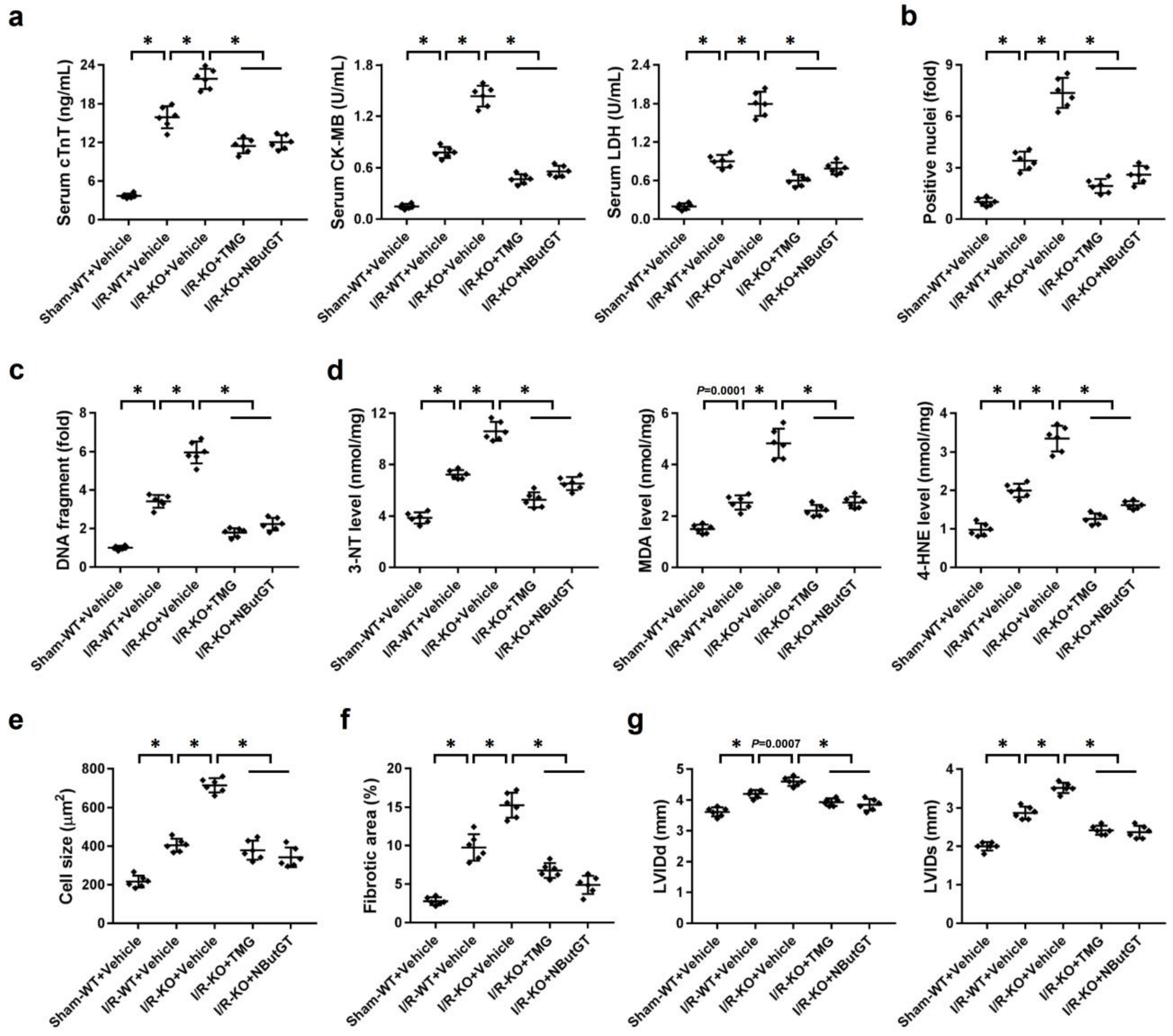

Supplementary Figure 12. OGA inhibition blocks cardiac I/R injury in Tisp40 KO mice in vivo. a, To inhibit OGA, Tisp40 KO mice were intraperitoneally injected with NButGT daily for 14 consecutive days or TMG every other day for 20 consecutive days, and the last injections of NButGT or TMG were done 30 min before cardiac I/R surgery. Next, circulating levels of cTnT, CK-MB and LDH were measured 4 h after I/R surgery (n=6). **b**, Quantitative results of TUNEL staining in the heart (n=6). **c**, DNA fragments in the heart (n=6). **d**, Levels of 3-NT, MDA and 4-HNE in the heart (n=6). **e-f**, Quantitative results of the cross-sectional area of cardiomyocyte and collagen deposition 4 weeks post-I/R surgery (n=6). **g**, Cardiac function was presented as LVIDd and LVIDs (n=6). All data are expressed as the mean ± S.D., and analyzed using one-way ANOVA followed by Tukey post hoc test. *$P < 0.0001$. Source data are provided as a Source Data file.

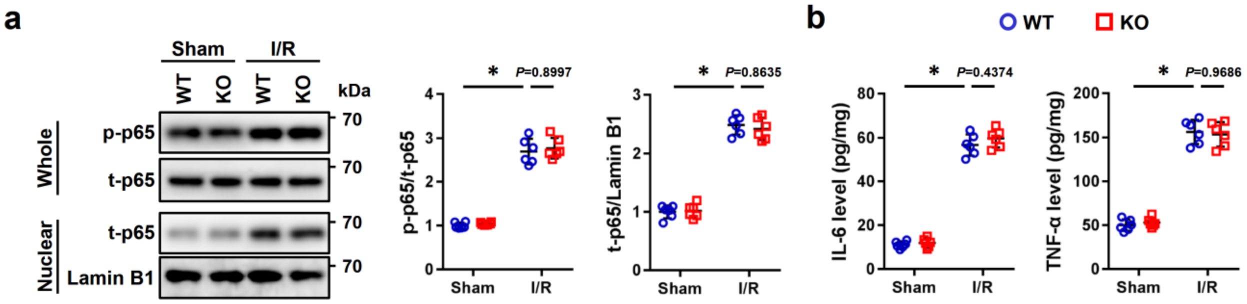

Supplementary Figure 13. Tisp40 deficiency does not affect NF-κB activation and cardiac inflammation in I/R-stressed hearts. a, Whole cell lysates and nuclear lysates from the heart were prepared for western blot (n=6). **b**, Levels of interleukin-6 (IL-6) and tumor necrosis factor-α (TNF-α) in the heart (n=6). All data are expressed as the mean ± S.D., and analyzed using one-way ANOVA followed by Tukey post hoc test. *P < 0.0001. Source data are provided as a Source Data file.

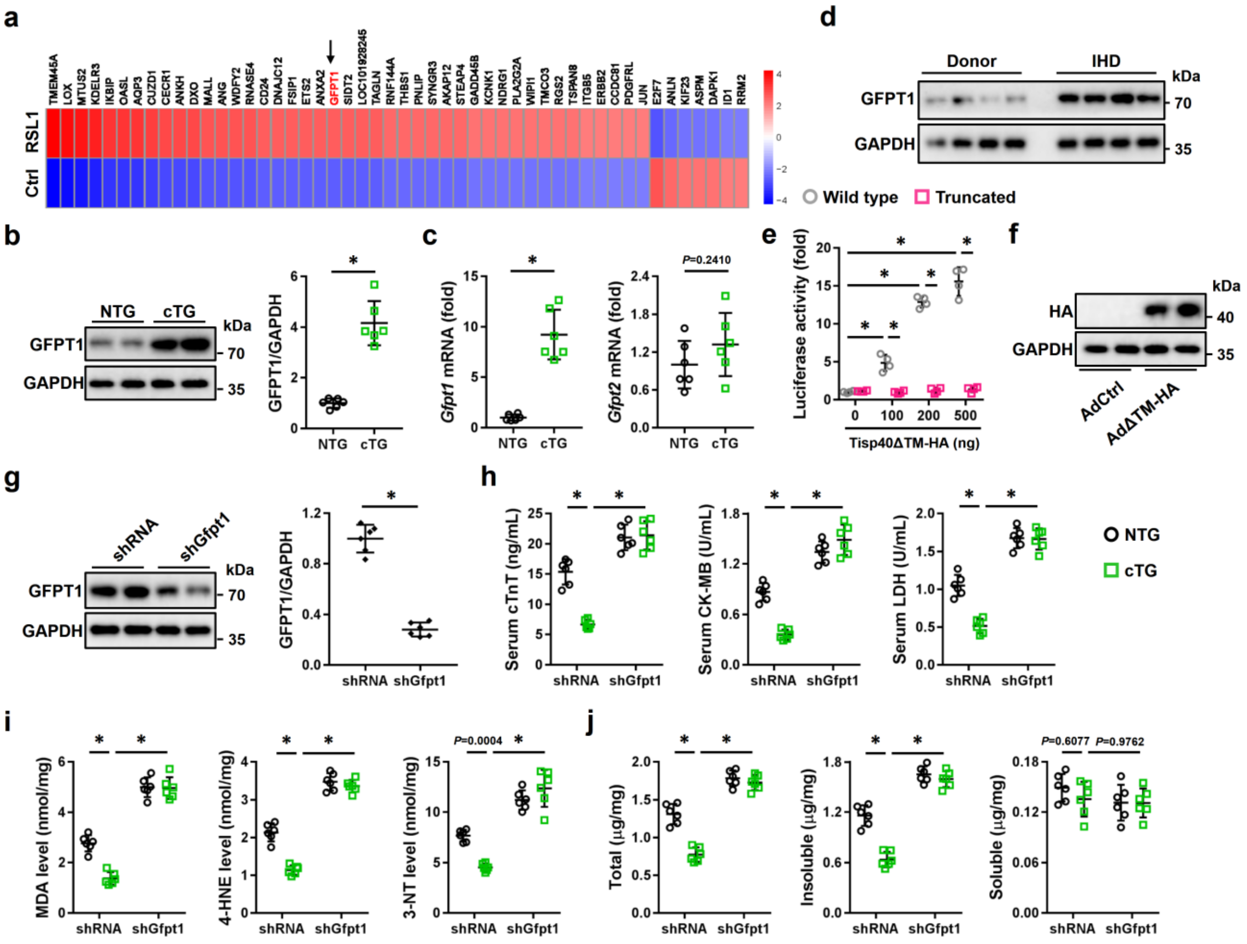

Supplementary Figure 14. GFPT1 silence abolishes the cardioprotective effects against I/R injury in Tisp40 cTG mice in vivo. **a**, Heatmap of GSE7223 dataset. **b-c**, Heart samples of Tisp40 cTG mice or NTG littermates were collected for western blot and quantitative real-time PCR (n=6). **d**, The left ventricles of ischemic heart disease (IHD) patients or donors were prepared for western blot (n=6). **e**, The plasmids containing wild type or truncated mouse GFPT1 promoter were co-transfected into HEK293T cells together with a mouse Tisp40ΔTM-HA plasmid for 48 h. The GFPT1 promoter activity was measured by a Dual-Luciferase reporter Assay system (n=4). **f**, Tisp40-deficient neonatal mouse cardiomyocytes were infected with AdΔTM-HA for 4 h and cultured in fresh medium for an additional 48 h. Next, cell lysates were prepared for western blot (n=6). **g**, Mice injected with shGfpt1 were maintained for 4 weeks, and then heart samples were collected for western blot (n=6). **h**, To knock down endogenous GFPT1 in the heart, Tisp40 cTG mice were intravenously injected with shGfpt1 4 weeks before I/R surgery. Next, circulating levels of cTnT, CK-MB and LDH were measured 4 h after I/R surgery (n=6). **i**, Levels of 3-NT, MDA and 4-HNE in the heart (n=6). **j**, Total, insoluble and soluble collagen content in the heart 4 weeks post-I/R surgery (n=6). All data are expressed as the mean ± S.D., and analyzed using one-way ANOVA followed by Tukey post hoc test. For the analysis in figure

S14b, c and g, an unpaired two-tailed Student′s *t*-test was conducted. $*P < 0.0001$. Source data are provided as a Source Data file.

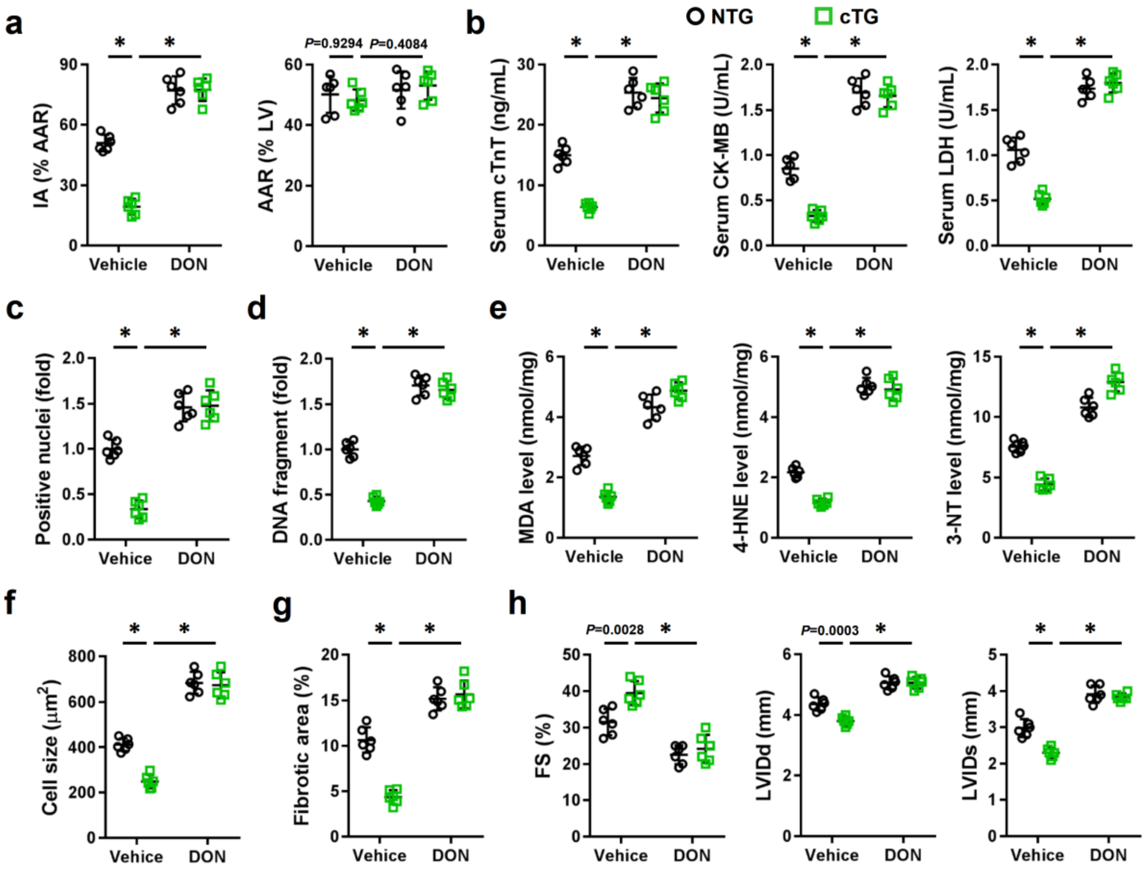

Supplementary Figure 15. GFPT1 inhibition abolishes the cardioprotective effects against I/R injury in Tisp40 cTG mice in vivo. a, To suppress GFPT1 activity, Tisp40 cTG mice were intraperitoneally injected with DON every other day for 20 consecutive days prior to-I/R surgery. Next, evans blue and TTC staining were performed to demarcate IA and AAR (n=6). **b**, Circulating levels of cTnT, CK-MB and LDH were measured 4 h after I/R surgery (n=6). **c**, Quantitative results of TUNEL staining in the heart (n=6). **d**, DNA fragments in the heart (n=6). **e**, Levels of 3-NT, MDA and 4-HNE in the heart (n=6). **f-g**, Quantitative results of the cross-sectional area of cardiomyocyte and collagen deposition 4 weeks post-I/R surgery (n=6). **h**, Cardiac function was presented as FS, LVIDd and LVIDs (n=6). All data are expressed as the mean ± S.D., and analyzed using one-way ANOVA followed by Tukey post hoc test. *P < 0.0001. Source data are provided as a Source Data file.

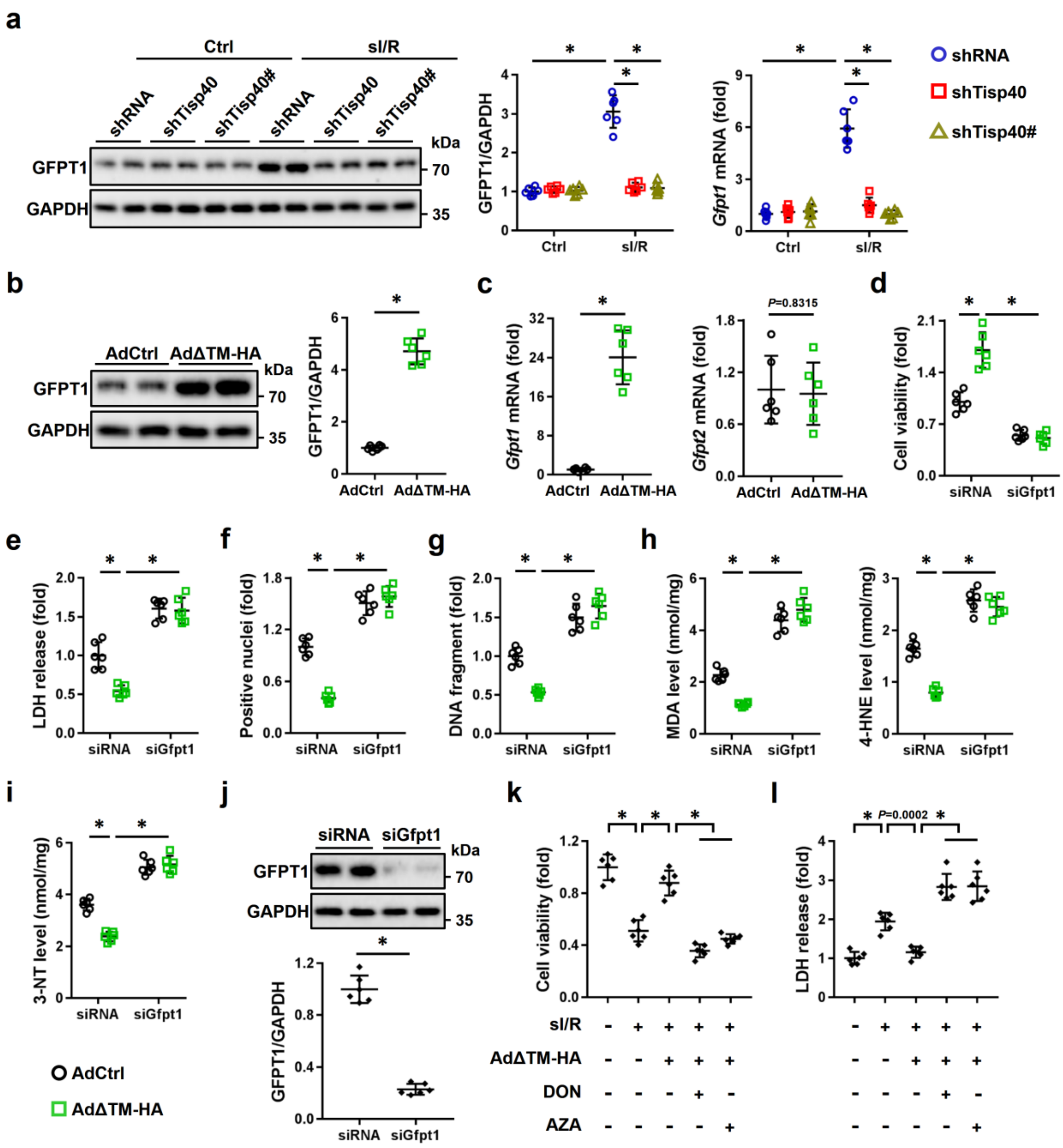

Supplementary Figure 16. GFPT1 inhibition abolishes Tisp40-mediated cellular protection against sI/R insult in vitro. a, Tisp40-silenced NRCMs with or without sI/R insult were collected for western blot and quantitative real-time PCR (n=6). **b-c**, Tisp40-overexpressed NRCMs were collected for western blot and quantitative real-time PCR (n=6). **d**, To knock down endogenous GFPT1 *in vitro*, NRCMs were pre-transfected with siGfpt1 for 4 h, followed by the incubation in fresh medium for an additional 24 h before Tisp40 manipulation. Next, cell viability was evaluated (n=6). **e**, LDH releases were calculated as (LDH level in ischemia medium + LDH in reperfusion medium)/(LDH in ischemia medium + LDH in reperfusion medium + LDH in cell lysate) (n=6). **f**, Quantitative results of TUNEL staining in NRCMs (n=6). **g**, DNA fragments in NRCMs (n=6). **h-i**, Levels of 3-NT, MDA and 4-HNE in NRCMs (n=6). **j**, NRCMs transfected with siGfpt1 or

siRNA were collected for western blot (n=6). **k**, To inhibit GFPT1 activity, DON or AZA was added to the medium during reperfusion, and then cell viability was evaluated (n=6). **l**, LDH releases were calculated as (LDH level in ischemia medium + LDH in reperfusion medium)/(LDH in ischemia medium + LDH in reperfusion medium + LDH in cell lysate) (n=6). All data are expressed as the mean ± S.D., and analyzed using one-way ANOVA followed by Tukey post hoc test. For the analysis in figure S16b, c and j, an unpaired two-tailed Student′s *t*-test was conducted. *$P < 0.0001$. Source data are provided as a Source Data file.

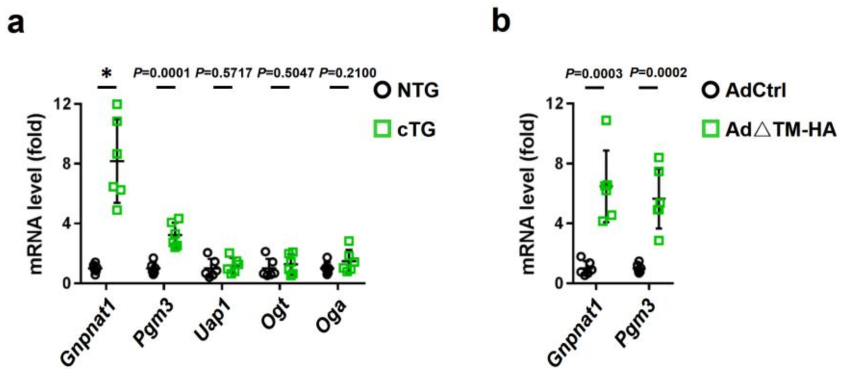

Supplementary Figure 17. Tisp40 overexpression increases the mRNA levels of GNPNAT1 and PGM3 in vivo and in vitro. a, Heart samples of Tisp40 cTG mice or NTG littermates were exposed to quantitative real-time PCR for the analysis of *Gnpnat1*, *Pgm3*, *Uap1*, *Oga* and *Ogt* mRNA levels (n=6). **b**, Tisp40-overexpressed NRCMs were collected for quantitative real-time PCR (n=6). All data are expressed as the mean ±S.D., and analyzed using an unpaired two-tailed Student′s *t*-test. *$P < 0.0001$. Source data are provided as a Source Data file.

Western blot images

Related to Figure 1B

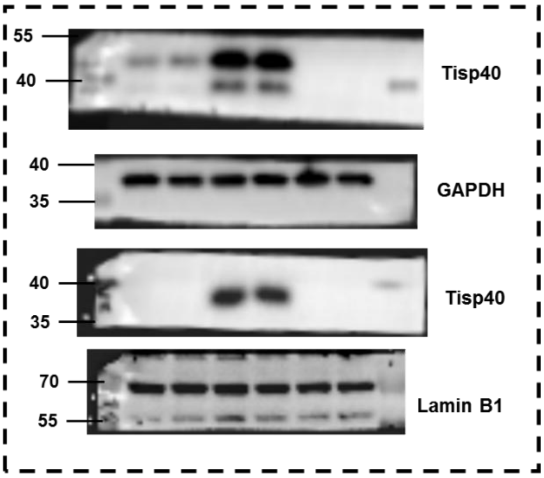

Related to Figure 1E

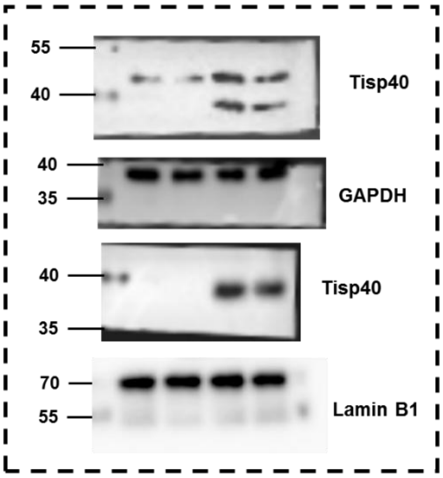

Related to Figure 1F

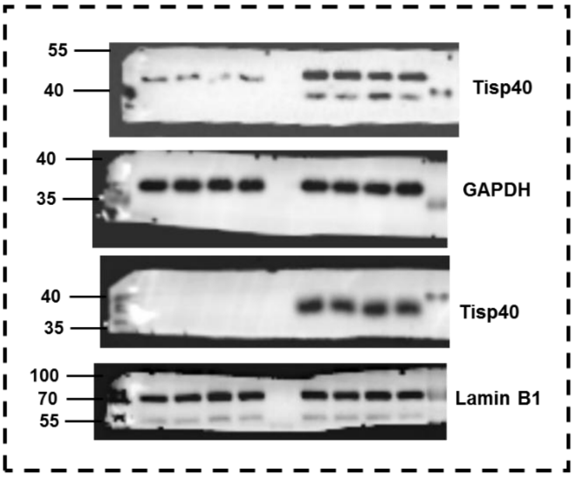

Related to Figure 1G

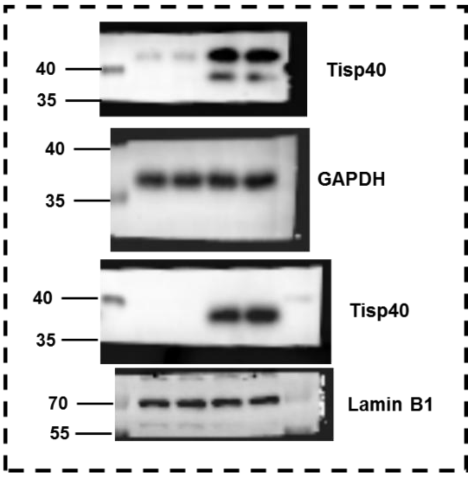

Related to supplementary Figure 3B

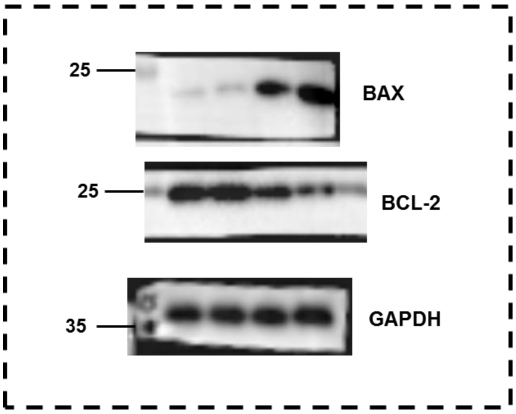

Related to supplementary Figure 4A

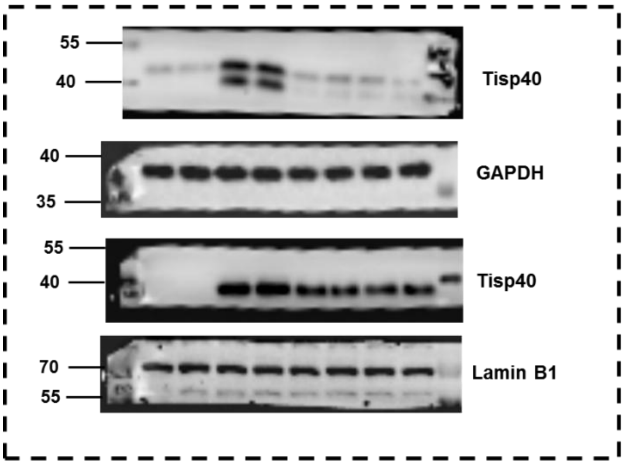

Related to supplementary Figure 4F

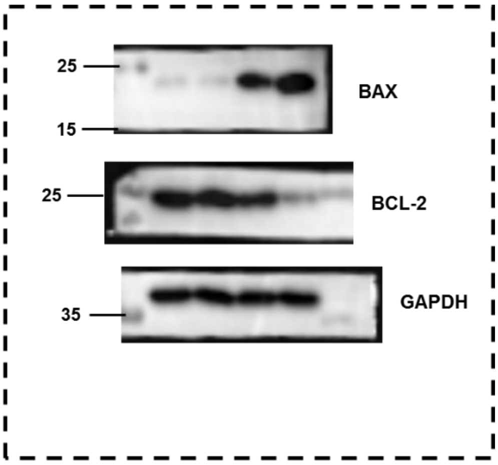

Related to supplementary Figure 8A

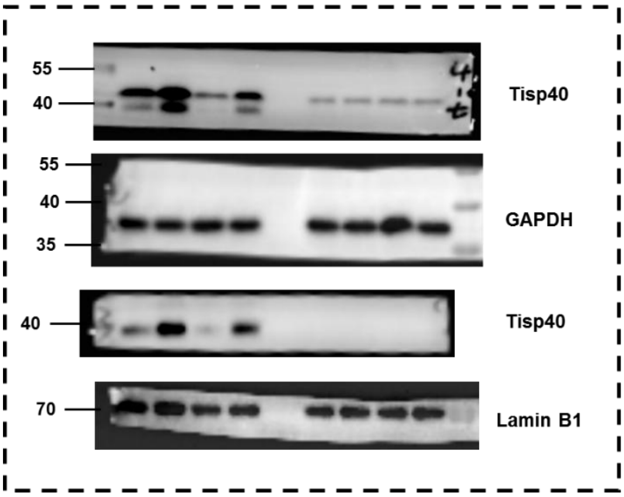

Related to supplementary Figure 8C

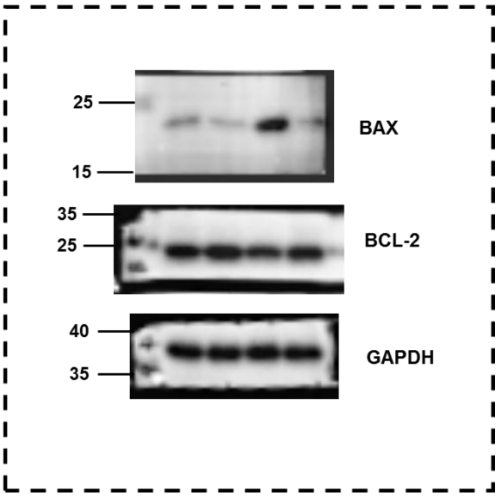

Related to Figure 5A

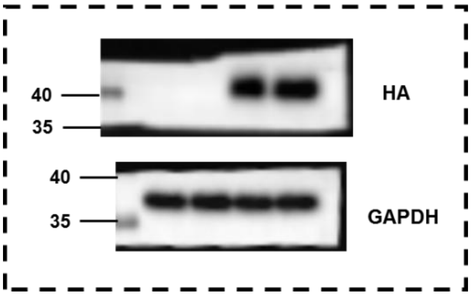

Related to supplementary Figure 9B

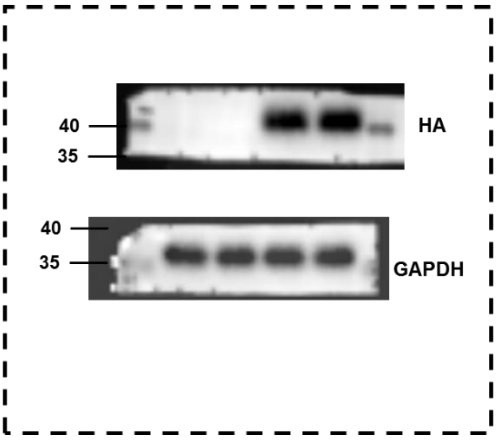

Related to supplementary Figure 9F

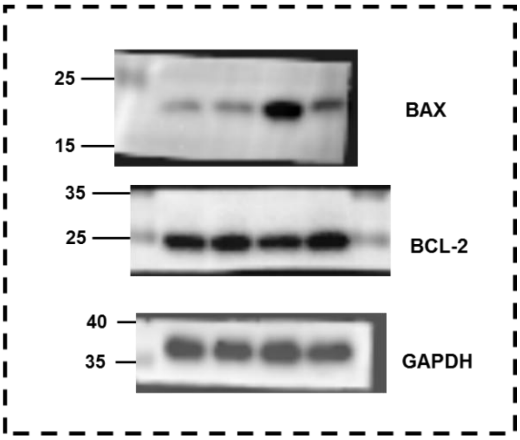

Related to Figure 6F

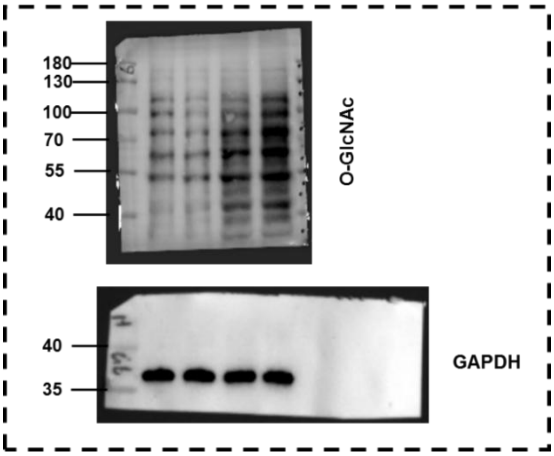

Related to supplementary Figure 10A

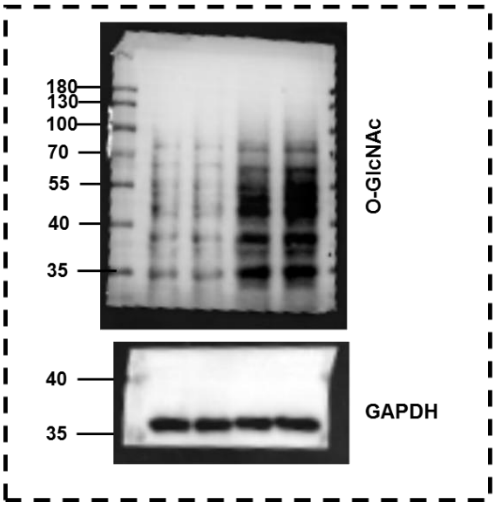

Related to supplementary Figure 10B

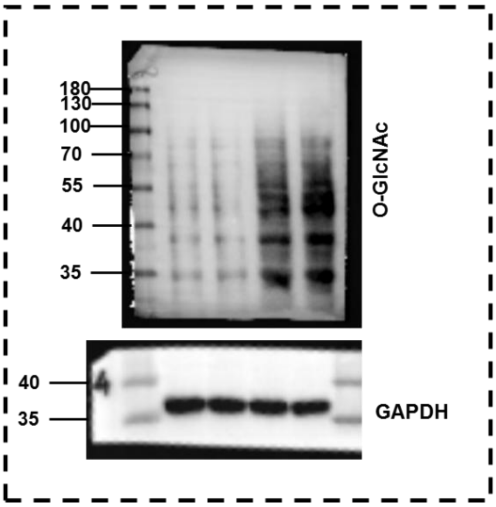

Related to supplementary Figure 13A

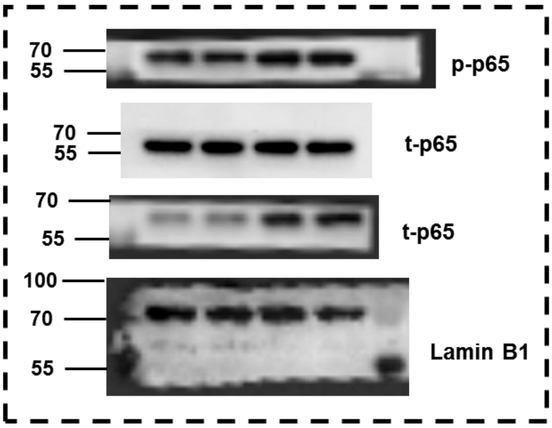

Related to Figure 7C

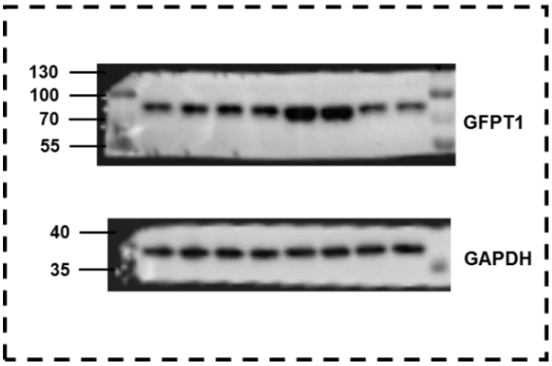

Related to Figure 7F

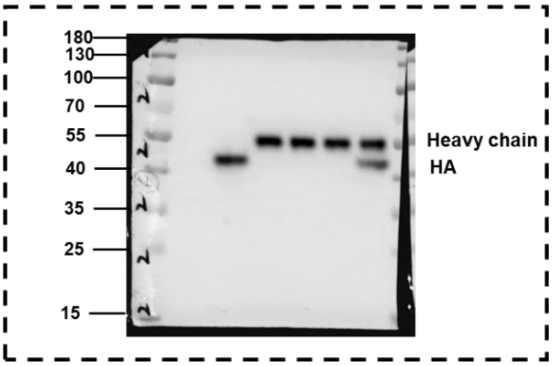

Related to supplementary Figure 14B

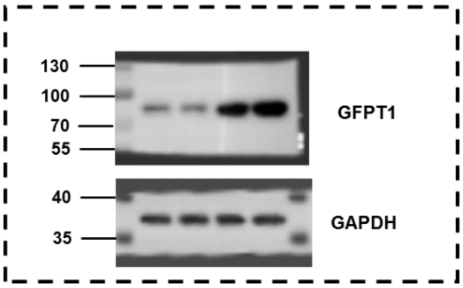

Related to supplementary Figure 14D

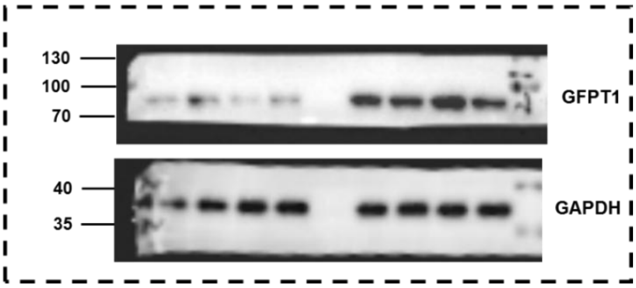

Related to supplementary Figure 14F

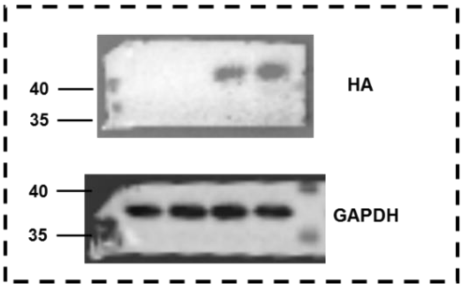

Related to supplementary Figure 14G

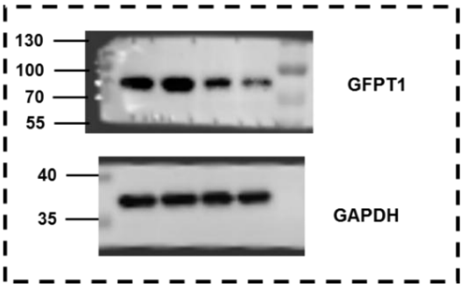

Related to supplementary Figure 16A

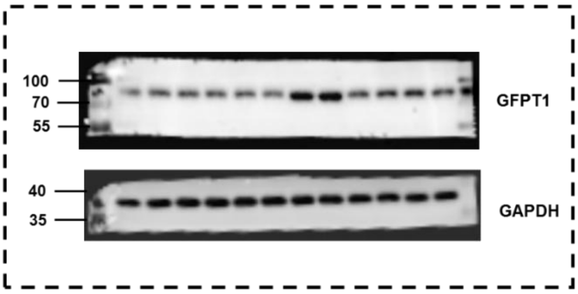

Related to supplementary Figure 16B

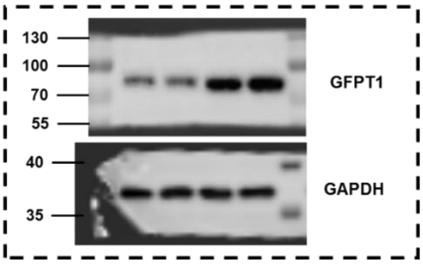

Related to supplementary Figure 16J

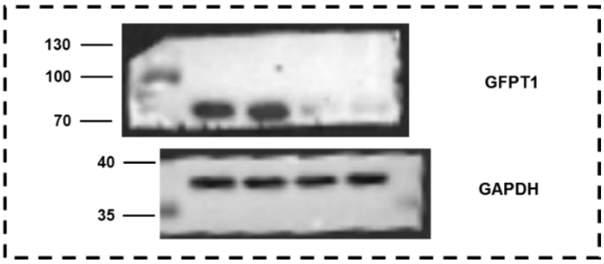

Related to Figure 8A

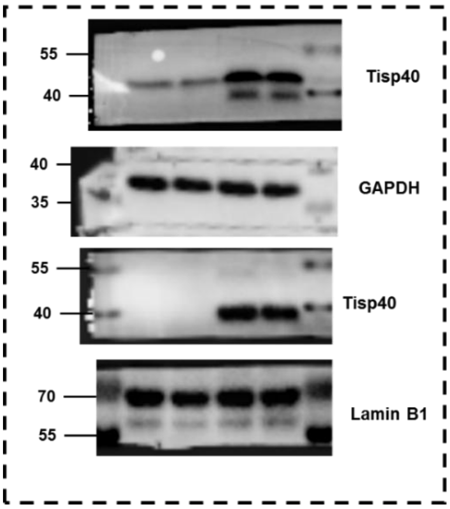

Related to Figure 8B

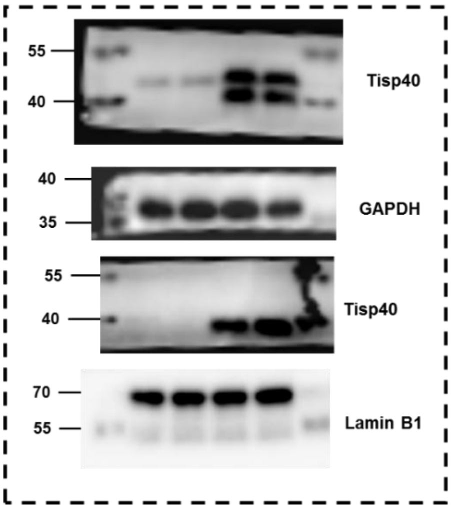

Related to Figure 8C

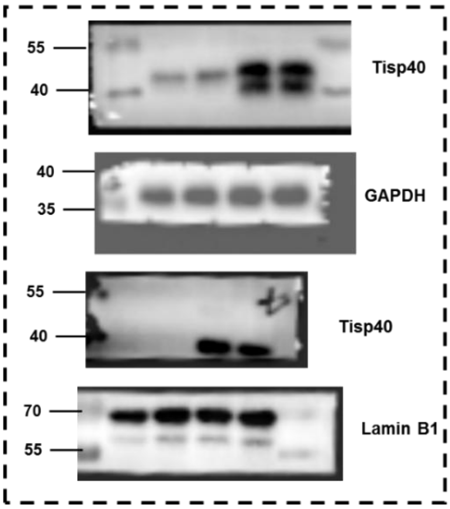

Related to Figure 8D

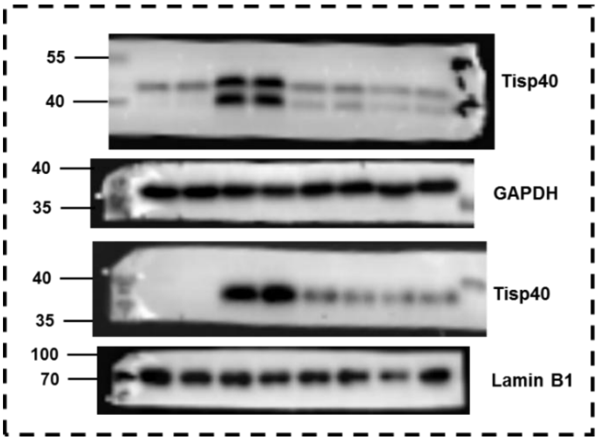

Related to Figure 8E

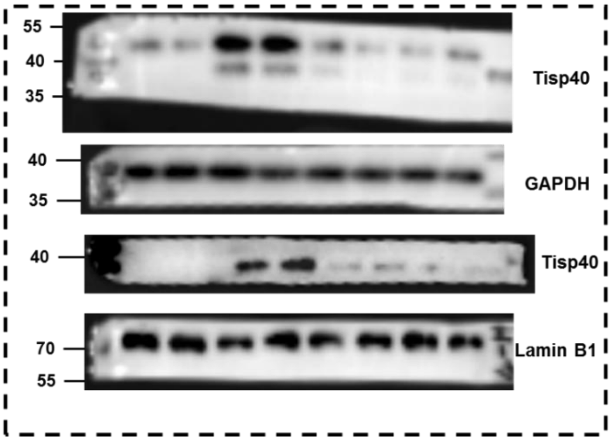

Supplement: Supplementary file 1 — Supplementary Information [file 41467_2023_39159_MOESM1_ESM.pdf]
