# Supplementary material for: Tisp40 prevents cardiac ischemia/reperfusion injury through the hexosamine biosynthetic pathway in male mice
Source: Nat Commun. 2023 Jun 8;14:3383. doi: 10.1038/s41467-023-39159-0 (PMC10250363; doi:10.1038/s41467-023-39159-0)
Supplement: Supplementary file 3 — Reporting Summary [file 41467_2023_39159_MOESM3_ESM.pdf]

nature portfolio

Corresponding author(s): Qi-Zhu Tang

Last updated by author(s): May 5, 2023

Reporting Summary

Nature Portfolio wishes to improve the reproducibility of the work that we publish. This form provides structure for consistency and transparency in reporting. For further information on Nature Portfolio policies, see our Editorial Policies and the Editorial Policy Checklist.

Statistics

For all statistical analyses, confirm that the following items are present in the figure legend, table legend, main text, or Methods section.

n/a	Confirmed	
☐	☒	The exact sample size (*n*) for each experimental group/condition, given as a discrete number and unit of measurement
☐	☒	A statement on whether measurements were taken from distinct samples or whether the same sample was measured repeatedly
☐	☒	The statistical test(s) used AND whether they are one- or two-sided<br>*Only common tests should be described solely by name; describe more complex techniques in the Methods section.*
☐	☒	A description of all covariates tested
☐	☒	A description of any assumptions or corrections, such as tests of normality and adjustment for multiple comparisons
☐	☒	A full description of the statistical parameters including central tendency (e.g. means) or other basic estimates (e.g. regression coefficient) AND variation (e.g. standard deviation) or associated estimates of uncertainty (e.g. confidence intervals)
☐	☒	For null hypothesis testing, the test statistic (e.g. *F*, *t*, *r*) with confidence intervals, effect sizes, degrees of freedom and *P* value noted<br>*Give P values as exact values whenever suitable.*
☒	☐	For Bayesian analysis, information on the choice of priors and Markov chain Monte Carlo settings
☒	☐	For hierarchical and complex designs, identification of the appropriate level for tests and full reporting of outcomes
☒	☐	Estimates of effect sizes (e.g. Cohen's *d*, Pearson's *r*), indicating how they were calculated

Our web collection on statistics for biologists contains articles on many of the points above.

Software and code

Policy information about availability of computer code

Data collection	Vevo® 3100 high resolution Preclinical Imaging System (FUJIFILM VisualSonics, Toronto, Canada), DP74 fluorescence microscope (OLYMPUS, Tokyo, Japan), ChemiDoc™ XRS+ System (Bio-Rad Laboratories, Inc.), Roche LightCycler 480 system, BioTek microplate reader (Winooski, Vermont, USA), ADVIA® 2400 automatic biochemical analyzer (Siemens Healthcare Diagnostics, Tarrytown, NY, USA), Agilent 1290 Infinity II series UHPLC System (Agilent Technologies, Palo Alto, CA, USA), Agilent 6495 Triple Quadrupole Mass Spectrometer (Agilent Technologies)
Data analysis	Image-Pro Plus 6.0 software (Media Cybernetics, Bethesda, MD, USA), Image Lab Software (version 6.0, Bio-Rad Laboratories, Inc.), GraphPad Prism (version 7.0), Excel

For manuscripts utilizing custom algorithms or software that are central to the research but not yet described in published literature, software must be made available to editors and reviewers. We strongly encourage code deposition in a community repository (e.g. GitHub). See the Nature Portfolio guidelines for submitting code & software for further information.

Data

Policy information about availability of data

All manuscripts must include a data availability statement. This statement should provide the following information, where applicable:

- Accession codes, unique identifiers, or web links for publicly available datasets
- A description of any restrictions on data availability
- For clinical datasets or third party data, please ensure that the statement adheres to our policy

The data that support the findings of this study are available within the main text, its Supplementary Information file and Source data. The microarray hybridization dataset is downloaded from the online Gene Expression Omnibus database (https://www.ncbi.nlm.nih.gov/geo/query/acc.cgi?acc=GSE7223)(Ben, A. S. et al 24). Source data are provided with this paper.

Human research participants

Policy information about studies involving human research participants and Sex and Gender in Research.

Reporting on sex and gender	Samples were collected from men and women depending on the availability of the heart explants, independently of the sex. Difference between male and female participants were not compared due to small group sizes. 9 men (5 for IHD patients) and 3 women (1 for IHD patients) were included in this study.
Population characteristics	All the patient's characteristics were described in our previous studies. 9 men (5 for IHD patients) and 3 women (1 for IHD patients) were included in this study. Age of the participants is between 28 and 74.
Recruitment	Patients were recruited according to the etiology and the severity of heart disease. Sex and age were random, and no self-selection bias or other biases existed.
Ethics oversight	Written informed consent was obtained from all patients and donors. All experimental procedures involving human samples in this study were in accordance with the Declaration of Helsinki, and also approved by the Review Board of Renmin Hospital of Wuhan University.

Note that full information on the approval of the study protocol must also be provided in the manuscript.

Field-specific reporting

Please select the one below that is the best fit for your research. If you are not sure, read the appropriate sections before making your selection.

☒ Life sciences ☐ Behavioural & social sciences ☐ Ecological, evolutionary & environmental sciences

For a reference copy of the document with all sections, see nature.com/documents/nr-reporting-summary-flat.pdf

Life sciences study design

All studies must disclose on these points even when the disclosure is negative.

Sample size	The sample size was determined based upon similar studies in this field by us and other groups (PMID: 24630721, 34536344, 35166002, 31209361), as being sufficient for quantification purposes, and upon variability observed in past experiments of similar nature.
Data exclusions	No data were excluded.
Replication	The detailed replication of each experiments has been provided in the Figure Legend, and all attempts at replication were successful yielding similar results.
Randomization	All samples were fully randomized to treatments throughout the study.
Blinding	The investigators were blinded to all grouping information in data collection and analysis.

Reporting for specific materials, systems and methods

We require information from authors about some types of materials, experimental systems and methods used in many studies. Here, indicate whether each material, system or method listed is relevant to your study. If you are not sure if a list item applies to your research, read the appropriate section before selecting a response.

Materials & experimental systems

n/a	Involved in the study
☐	☒ Antibodies
☐	☒ Eukaryotic cell lines
☒	☐ Palaeontology and archaeology
☐	☒ Animals and other organisms
☒	☐ Clinical data
☒	☐ Dual use research of concern

Methods

n/a	Involved in the study
☒	☐ ChIP-seq
☒	☐ Flow cytometry
☒	☐ MRI-based neuroimaging

Antibodies

Antibodies used

α-actinin Abcam ab68167 IF (1:100); α-SMA Abcam ab5694 IHC (1:100); BAX CST 2772 WB (1:1000); BCL-2 Abcam ab196495 WB (1:1000); GAPDH CST 2118 WB (1:1000); GFPT1 Proteintech 14132-1-AP WB (1:500), IF (1:100); HA-Tag CST 3724 WB (1:2000), IF (1:200), ChIP (1:50); KDEL Abcam ab176333 IF (1:100); Lamin B1 Abcam ab16048 WB (1:1000); O-GlcNAc (CTD110.6) CST 9875 WB (1:1000), IF (1:100); Tisp40 Santa Cruz sc-390842 WB (1:500), IF (1:100); p-p65 CST 3033 WB (1:1000); t-p65 CST 8242 WB (1:1000);goat anti-mouse IgG Alexa Fluor 488 secondary antibodies Invitrogen A11001 IF (1:200); goat anti-rabbit IgG Alexa Fluor 568 secondary antibodies Invitrogen A11011 IF (1:200).

Validation

All antibodies in this study were used and validated according to the provided data sheets and references for the specific technique (western blot, immunostaining or ChIP) found directly on the manufacturer's website.
1. α-actinin: https://www.abcam.cn/products/primary-antibodies/sarcomeric-alpha-actinin-antibody-ep2529y-ab68167.html
2. α-SMA: https://www.abcam.cn/products/primary-antibodies/alpha-smooth-muscle-actin-antibody-ab5694.html
3. BAX: https://www.cellsignal.cn/products/primary-antibodies/bax-antibody/2772?site-search-type=Products&N=4294956287&Ntt=2772&fromPage=plp&_requestid=162345
4. BCL-2: https://www.abcam.cn/products/primary-antibodies/bcl-2-antibody-ab196495.html
5. GAPDH: https://www.cellsignal.cn/products/primary-antibodies/gapdh-14c10-rabbit-mab/2118?site-search-type=Products&N=4294956287&Ntt=2118&fromPage=plp&_requestid=162510
6. GFPT1: https://www.ptgcn.com/products/GFPT1-Antibody-14132-1-AP.htm
7. HA-Tag: https://www.cellsignal.cn/products/primary-antibodies/ha-tag-c29f4-rabbit-mab/3724?site-search-type=Products&N=4294956287&Ntt=3724&fromPage=plp&_requestid=162618
8. KEDL: https://www.abcam.cn/products/primary-antibodies/kdel-antibody-epr12668-ab176333.html
9. Lamin B1: https://www.abcam.cn/products/primary-antibodies/lamin-b1-antibody-nuclear-envelope-marker-ab16048.html
10. O-GlcNAc: https://www.cellsignal.cn/products/primary-antibodies/o-glcnac-ctd110-6-mouse-mab/9875?site-search-type=Products&N=4294956287&Ntt=9875&fromPage=plp&_requestid=163104
11. Tisp40: https://www.scbt.com/p/creb3l4-antibody-c-11?requestFrom=search
12. p-p65: https://www.cellsignal.cn/products/primary-antibodies/phospho-nf-kb-p65-ser536-93h1-rabbit-mab/3033?site-search-type=Products&N=4294956287&Ntt=3033&fromPage=plp&_requestid=163229
13. t-p65: https://www.cellsignal.cn/products/primary-antibodies/nf-kb-p65-d14e12-xp-rabbit-mab/8242?site-search-type=Products&N=4294956287&Ntt=8242&fromPage=plp&_requestid=163261
14. goat anti-mouse IgG Alexa Fluor 488 secondary antibodies: https://www.thermofisher.cn/cn/zh/antibody/product/Goat-anti-Mouse-IgG-H-L-Cross-Adsorbed-Secondary-Antibody-Polyclonal/A-11001
15. goat anti-rabbit IgG Alexa Fluor 568 secondary antibodies: https://www.thermofisher.cn/cn/zh/antibody/product/Goat-anti-Rabbit-IgG-H-L-Cross-Adsorbed-Secondary-Antibody-Polyclonal/A-11011

Eukaryotic cell lines

Policy information about cell lines and Sex and Gender in Research

Cell line source(s)	HEK293T cells were obtained from the Type Culture Collection of the Chinese Academy of Sciences, Shanghai, China.
Authentication	Before the experiments, all cell lines were verified through short tandem repeat DNA profiling.
Mycoplasma contamination	Mycoplasma contamination was checked, and the test results were negative.
Commonly misidentified lines (See ICLAC register)	No commonly misidentified cells were used in this study.

Animals and other research organisms

Policy information about studies involving animals; ARRIVE guidelines recommended for reporting animal research, and Sex and Gender in Research

Laboratory animals

Global Tisp40 knockout (KO) mice (stock No. RBRC01942) were purchased from the RIKEN BioResource Research Center and backcrossed to C57BL/6 strain over 10 generations. To establish cardiomyocyte-restricted Tisp40 transgenic (cTG) mice, full-length mouse Tisp40 cDNA was cloned under control of the cardiac α-myosin heavy chain (α-MHC) promoter, which was then microinjected into fertilized mouse embryos as we previously described. These mice were kept in a specific pathogen free, environmentally suitable barrier system at the Cardiovascular Research Institute of Wuhan University with free access to standard food and water. To induce

cardiac I/R injury in vivo, male mice aged 10-12 weeks were used. To isolate neonatal rat cardiomyocytes (NRCMs), 1-3-day-old Sprague-Dawley rats were used as we previously described.
These mice were kept in Individually Ventilated Cages with the density of 4-6 mice per cage under a specific pathogen free, environmentally suitable barrier system (20-25 °C and 45-55% humidity) on a regular 12 h light/dark cycle at the Cardiovascular Research Institute of Wuhan University. All mice were fed with a irradiated chow diet (#1035 for reproductive feeding and #1025 for maintenance feeding, Beijing HFK Bioscience Co., Ltd, Beijing, China), with free access to drinking water. All mice were sacrificed at indicating times by cervical dislocation.

Wild animals	No wild animals were used in the study.
Reporting on sex	Several studies showed that risk factors and adaptations of ischemic cardiac injury and heart failure in men and women are different. Pre-menopausal women are better protected against ischemic cardiac injury and exhibit better outcome compared with men. Moreover, maladaptive left ventricular remodelling occurs more frequently in men and is associated with greater activation of profibrotic and inflammatory markers. Collectively, we focused more attention on ischemic cardiac injury in male mice. In addition, it has been extensively reported that male mice are more susceptible to develop ischemic cardiac injury than females (24630721, 34536344). Neonatal Sprague-Dawley rats with 1-3 days old were used for the isolation of NRCMs regardless of the sex, as it is difficult to distinguish the sex of these neonatal rats
Field-collected samples	No field collected samples were used in the study.
Ethics oversight	All the experimental procedures were approved by the Animal Care and Use Committee of Renmin Hospital of Wuhan University, and were also in accordance with the Guidelines for the Care and Use of Laboratory Animals published by the US National Institutes of Health.

Note that full information on the approval of the study protocol must also be provided in the manuscript.
